# An epilepsy-associated mutation in the nuclear import receptor KPNA7 reduces nuclear localization signal binding

**DOI:** 10.1038/s41598-020-61369-5

**Published:** 2020-03-16

**Authors:** Luke T. Oostdyk, Zhenjia Wang, Chongzhi Zang, Hui Li, Michael J. McConnell, Bryce M. Paschal

**Affiliations:** 1 Department of Biochemistry & Molecular Genetics, University of Virginia School of Medicine, Charlottesville, VA 22908 USA; 2 Center for Cell Signaling, University of Virginia School of Medicine, Charlottesville, VA 22908 USA; 3 Center for Public Health Genomics and Department of Public Health Sciences, University of Virginia School of Medicine, Charlottesville, VA 22908 USA; 4 Department of Pathology, University of Virginia School of Medicine, Charlottesville, VA 22908 USA; 5 Center for Brain Immunology and Glia, University of Virginia School of Medicine, Charlottesville, VA 22908 USA; 6 Department of Neuroscience, University of Virginia School of Medicine, Charlottesville, VA 22908 USA

**Keywords:** Biochemistry, Molecular biology

## Abstract

KPNA7 is a member of the Importin-*α* family of nuclear import receptors. KPNA7 forms a complex with Importin-*β* and facilitates the translocation of signal-containing proteins from the cytoplasm to the nucleus. Exome sequencing of siblings with severe neurodevelopmental defects and clinical features of epilepsy identified two amino acid-altering mutations in KPNA7. Here, we show that the E344Q substitution reduces KPNA7 binding to nuclear localization signals, and that this limits KPNA7 nuclear import activity. The P339A substitution, by contrast, has little effect on KPNA7 binding to nuclear localization signals. Given the neuronal phenotype described in the two patients, we used SILAC labeling, affinity enrichment, and mass spectrometry to identify KPNA7-interacting proteins in human induced pluripotent stem cell-derived neurons. We identified heterogeneous nuclear ribonucleoproteins hnRNP R and hnRNP U as KPNA7-interacting proteins. The E344Q substitution reduced binding and KPNA7-mediated import of these cargoes. The c.1030G > C allele which generates E344Q is within a predicted CTCF binding site, and we found that it reduces CTCF binding by approximately 40-fold. Our data support a role for altered neuronal expression and activity of KPNA7 in a rare type of pediatric epilepsy.

## Introduction

The bi-directional transport of proteins and RNAs between the cytoplasm and nucleus is one of many pathways that contribute to cellular homeostasis. Dysregulation of nuclear transport has been implicated in diseases including cancer and diverse neurodegenerative disorders^1,2^. This includes disruption of pathways responsible for facilitating nuclear import of proteins that contain a nuclear localization signal (NLS). The composition and sequence of NLSs vary between proteins but consist primarily of either one (monopartite) or two (bipartite) clusters of amino acids enriched in the basic residues lysine and arginine^3,4^. Classical NLS import is mediated by Importin *α* (Imp-*α*) and Importin *β* (Imp-*β*)^5^. Imp-*α* functions as an adapter and makes direct contact with both an NLS sequence and Imp-*β* to facilitate the assembly of a heterotrimeric import complex that shuttles from the cytoplasm to the nucleus^6^. Within the nucleus, RanGTP facilitates release of the NLS containing proteins and it also promotes recycling of the import machinery to the cytoplasm, in preparation for a new round of import^7,8^.

In humans, the Imp-*α* family, which is also known as the karyopherin *α* family, contains seven members (KPNA1-KPNA7)^9^. These receptors have a highly related protein architecture that includes an N-terminal Imp-*β* binding (IBB) domain and a super-helical core structure formed by the tandem arrangement of ten armadillo (ARM) repeats^10,11^. A subset of the ARMs are used to create the surfaces for NLS binding. ARMs 2–4 provide the major NLS binding groove for both mono- and bipartite NLS sequences, while ARMs 6–8 generate the minor NLS binding groove for binding the smaller, second cluster of basic residues in bipartite NLSs^12^. Despite the overall similarity in tertiary structure and the high degree of amino acid conservation in the NLS-binding grooves, KPNA proteins are capable of discriminating between certain NLSs^13,14^.

NLS cargo specificity, combined with differential expression of Imp-*α* isoforms is utilized in developmental programs across species^14^. Examples include germ cell maturation in Drosophila and mice^15–17^, and neuronal development in mice^18^. Aberrant expression of individual Imp-*α* isoforms has been suggested to play roles in diseases, including cancer^19–21^, neurodegenerative disorder^22–25^ and inflammatory bowel disease^26^. These data suggest that the relative expression levels of KPNA proteins are important for maintaining cellular homeostasis. This presumably relates to the fact that at least some NLS cargo proteins are preferentially transported by select Imp-*α* isoforms^27^.

KPNA7 is the most recently identified and the most divergent member of the Imp-*α* family in humans^9^. It shares 55% amino acid identity with its closest related isoform, KPNA2^9^. In multiple species, expression of KPNA7 orthologs is mainly limited to the ovary, oocyte and developing embryo^28–31^. In humans, high KPNA7 expression has been observed in pancreatic cancers as a result of gene duplication events^32^. Additionally, compound heterozygous mutations in KPNA7 have been associated with neurodevelopmental defects including severe developmental disability, infantile spasms, and epilepsy^23^. The two mutations (c.1015C > G and c.1030G > C) both result in amino acid substitutions (P339A and E344Q) in the seventh ARM repeat of the protein, proximal to the minor NLS binding groove, but how these amino acid changes affect KPNA7 protein activity has not been directly examined. The association of KPNA7 mutations with neuronal defects, and its expression in early development, suggests that KPNA7 might make critical contributions to the development of the nervous system.

In this study, we found that KPNA7 expression is induced during neuronal differentiation of human induced pluripotent stem cells (iPSCs). We utilized iPSC-derived neurons, SILAC labeling, and mass spectrometry to identify hnRNP R and hnRNP U as KPNA7 interacting proteins. In permeabilized cell transport assays, KPNA7 binds and facilitates nuclear import of the bipartite NLS in hnRNP R and the monopartite NLS sequence in hnRNP U. The epilepsy-associated substitution, E344Q, reduces KPNA7 binding and nuclear import mediated by the NLS in hnRNP R and hnRNP U. Lastly, the DNA mutation (c.1030G > C) that generates the E344Q substitution maps to a CTCF binding site, and using fluorescence anisotropy, we determined that the mutation reduces CTCF binding ~40-fold. Our data provide insight into how a single base change can affect both the activity and expression of a transport receptor.

## Results

### The amino acid substitution E344Q in KPNA7 reduces NLS binding and import

**Figure 1:**
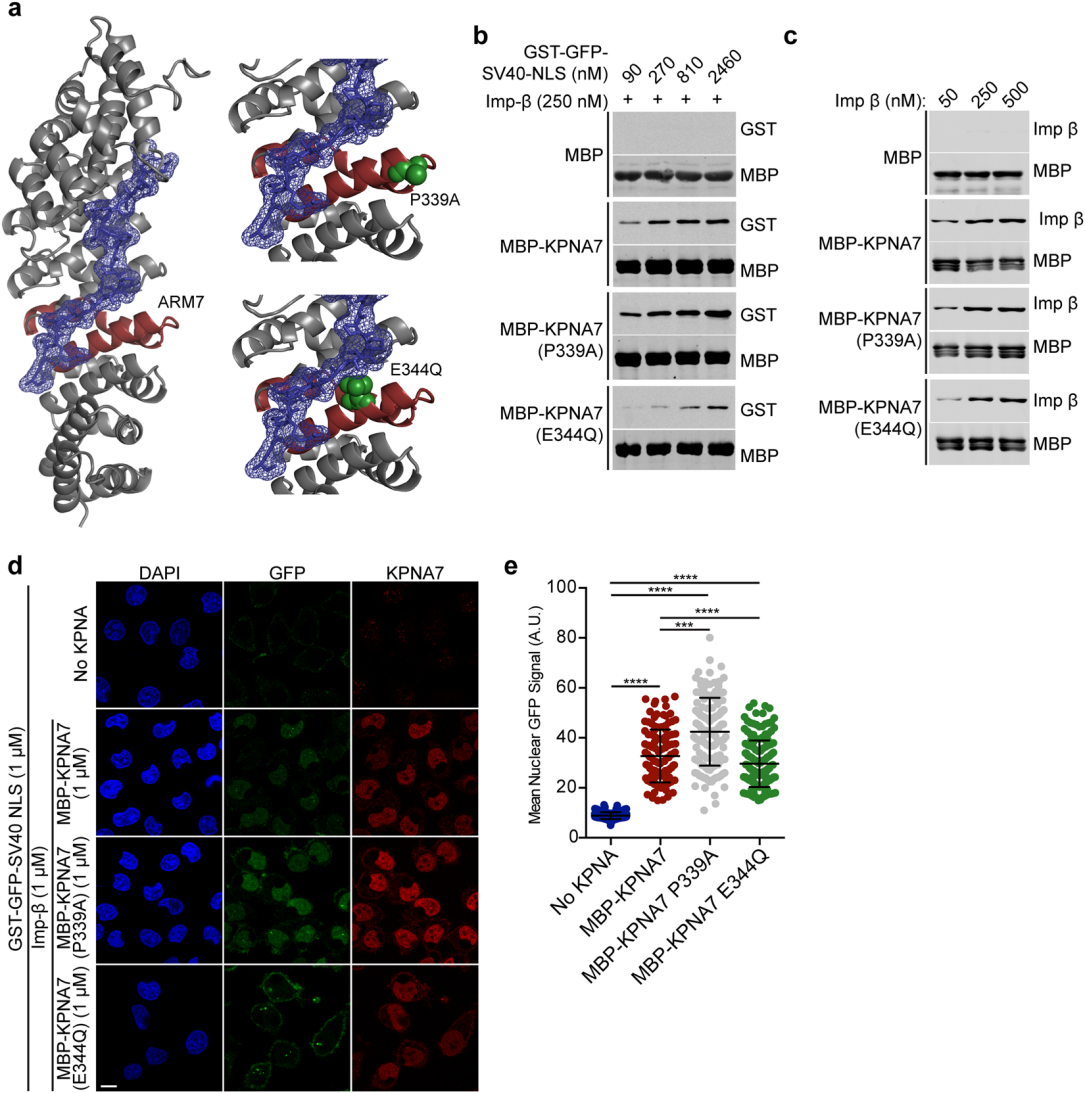
The amino acid substitution E344Q in ARM7 reduces KPNA7 binding to the SV40 NLS and reduces import in permeabilized cells. (**a**) Structural model of KPNA7 generated by threading the KPNA7 primary sequence onto the structure of KPNA2 (PBD: 1PJM) using SWISS-MODEL software. The bipartite NLS sequence from Rb is shown in wireframe (blue), with the electron surface density shown in blue surface mesh. The 7^*th*^ ARM repeat is highlighted (red). Insets show the P339A and E344Q amino acid substitutions in space-fill (green). Images were rendered in PyMol. (**b**) Binding assay with the indicated MBP fusion protein immobilized on amylose resin and incubated with GST-GFP-SV40-NLS at the indicated concentrations and Imp-*β* (250 nM). (**c**) Binding assay with the indicated MBP fusion proteins immobilized on amylose resin and incubated with the indicated concentrations of Imp-*β*. Blots in **b,c** were cropped for presentation (see Supplementary Information for full images). (**d**) Fluorescence microscopy images of permeabilized cell import assays showing the nuclear accumulation of GST-GFP-SV40 NLS. The assays were performed in HeLa cells permeabilized with 0.005% digitonin, and incubated with Ran (0.2 *μ*M), NTF2 (2 *μ*M) and an energy regenerating system with the recombinant factors indicated. Confocal IF microscopy was used to visualize. (**e**) Quantification of SV40-NLS import for each reaction. At least 100 cells of each were quantified. (Scale: 10 *μ*m. ****p < 0.0001, ***p < 0.001).

By protein modeling, the amino acid substitutions P339A and E344Q in KPNA7 (based on WGS data from siblings with epilepsy^23^) map to ARM7 near the predicted NLS binding groove (Fig. 1a). The proximity of these amino acid substitutions raised the possibility they could affect NLS binding (positively or negatively). We tested this by immobilizing WT and mutant forms of MBP-KPNA7 on maltose beads and performing binding assays with recombinant GST-SV40-NLS. A saturating concentration of Imp-*β* was included to minimize the potential contribution of auto-inhibition, which in the absence of Imp-*β* would reduce NLS binding. We found that SV40-NLS binding to KPNA7 was reduced slightly by the E344Q substitution relative to both the WT and P339A substitution (Fig. 1b). The level of Imp-*β* binding to all three KPNA7 proteins was similar, indicating mutant proteins immobilized on a bead surface are functional for protein-protein interactions (Fig. 1c). To determine if the mutations affect nuclear import mediated by KPNA7, we performed digitonin-permeabilized cell import assays using recombinant KPNA7 proteins and GST-GFP-NLS as the import substrate. We also processed the cells for detection of KPNA7 by immunofluorescence microscopy. Nuclear import of GST-GFP-NLS was dependent on addition of recombinant import factors (Fig 1d,e). We observed a small reduction in nuclear import of GST-GFP-NLS by KPNA7 (E344Q) compared to wild-type KPNA7 (~20% reduction, p < 0.0001). By immunofluorescence microscopy, nuclear accumulation of KPNA7(E344Q) was comparable to that of WT KPNA7 (Fig. 1d), consistent with the similar levels of Imp-*β* binding to the proteins (Fig. 1b).

**Figure 2:**
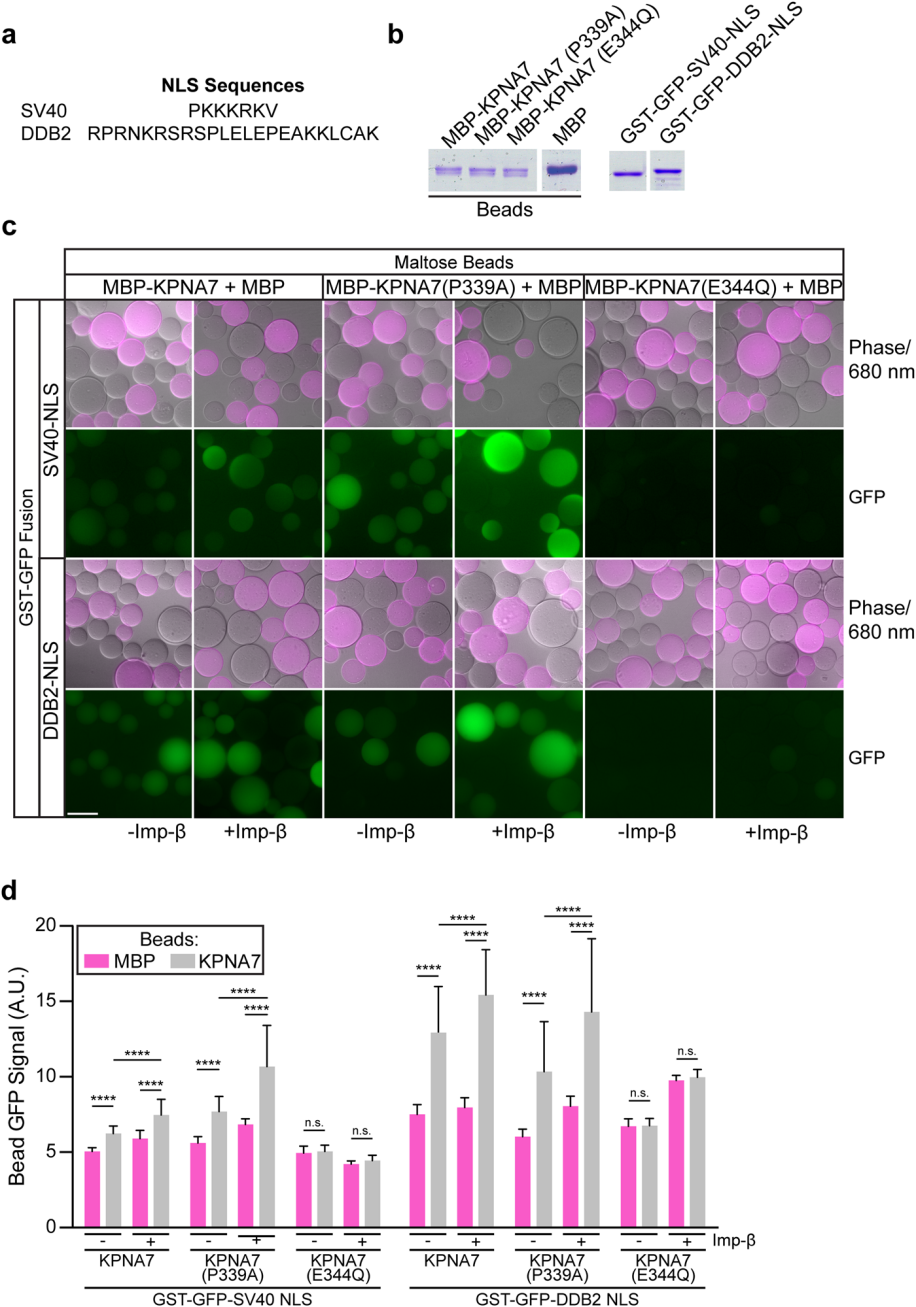
The E344Q substitution in KPNA7 reduces binding to mono- and bipartite NLS sequences as determined by bead-based fluorescence imaging. (**a**) NLS sequences from SV40 and DDB2. (**b**) Amylose beads (0.5 *μ*l per lane) used for fluorescence binding assay analyzed by SDS-PAGE and Coomassie blue (CB) staining. Gel images cropped for presentation; full gel image is available in Supplementary Materials. (**c**) Bead-based fluorescence imaging assay of MBP-KPNA7 proteins to the indicated NLS sequence. MBP-KPNA7 beads were combined with control MBP beads with a tracer amount of IRDye680-labeled MBP. Incubations were performed with the GST-GFP NLS constructs (750 nM) ± Imp-*β* (1 *μ*M), washed and imaged. (**d**) Fluorescence intensity of GFP was measured for MBP beads (680 nm positive) and MBP-KPNA7 beads (680 nm negative), and mean values were plotted. At least 30 beads of each condition were measured. (Scale: 100 *μ*M, ****p < 0.0001).

**Figure 3:**
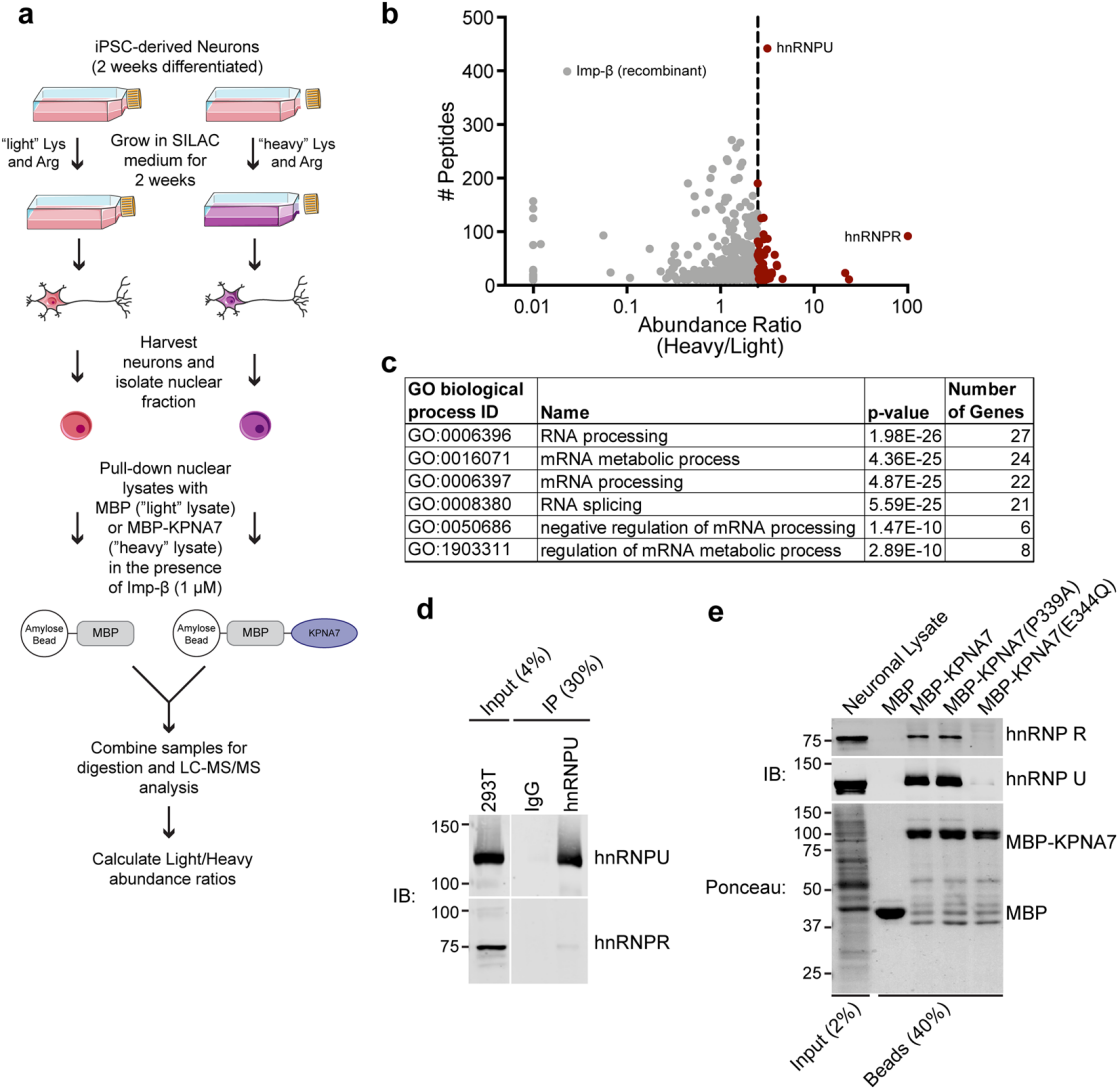
SILAC-Mass Spectrometry identifies hnRNP R and hnRNP U as KPNA7 interacting proteins. (**a**) Workflow for SILAC-mass spectrometry to identify nuclear KPNA7 interacting proteins from iPSC-derived neurons. (**b**) Plot showing the abundance ratio (measure of the enrichment for the heavy KPNA7 pull-down sample) versus the number of peptides identified from a given protein. A vertical line is drawn at abundance ratio of 2.75 and proteins with greater than this value are shown in red. (**c**) Gene ontology (GO) biological process enrichment analysis was performed on the 41 genes with greater than 10 identified peptides and abundance ratios greater than 2.75. (**d**) Immunoprecipitation of hnRNP U from HEK293T cells. Co-IP of hnRNP R was analyzed by western blot. (**e**) Validation of two candidate binding partners, hnRNP R and hnRNP U. MBP and MBP-KPNA7 proteins were immobilized on amylose resin and used in pull-downs with neuronal lysates from 4-week old 9429A hiPSC-derived neurons. Binding of hnRNP R and hnRNP U was analyzed by western blot. Blots in d and e were cropped for presentation; complete blot images are in the Supplementary Materials. This figure utilizes images adapted from Servier Medical Art (https://smart.servier.com) which are licensed under a Creative Commons Attribution 3.0 Unported License (https://creativecommons.org/licenses/by/3.0/legalcode).

To further characterize the effects of the mutations on KPNA7 function, we utilized a bead-based fluorescent assay to visualize and measure NLS binding to import receptors. The assay enables detection of protein binding to a protein partner immobilized on the bead surface in a timescale of minutes. The “bead halo assay” was used originally by Rexach and coworkers to study nucleoporin interactions^33^. We modified the assay setup to include negative control beads that are marked with a tracer amount of fluorescently-labeled (IRDye-680) MBP. This allows the negative control beads (pink) and MBP-KPNA7 beads (detected by phase-contrast) to be combined in the same reaction. The fluorescent MBP protein allows the negative control beads to be clearly distinguished from the MBP-KPNA7 beads (Fig. 2c). We immobilized each KPNA7 protein (1 *μ*g/*μ*l beads) and confirmed that comparable amounts were immobilized by SDS PAGE and Coomassie blue staining (Fig. 2b). To visualize binding, we used recombinant GST-GFP-tagged proteins that contain the monopartite NLS from SV40 and the bipartite NLS from DDB2 (Fig. 2a,b). Binding was quantified by fluorescence microscopy and statistical comparisons were made between the MBP beads and MBP-KPNA7 beads. We found that the SV40 NLS and the DDB2 NLS each bound to MBP-KPNA7 WT and MBP-KPNA7(P339A) proteins, and binding in these reactions was increased slightly by addition of Imp-*β* (Fig. 3d). In contrast, the level of binding of the SV40 NLS and the DDB2 NLS to MBP-KPNA7(E344Q) beads was indistinguishable from binding to control beads in the same reaction that were marked with fluorescent MBP. Imp-*β* addition resulted in enhanced binding to WT and P339A KPNA7, but not to E344Q KPNA7. We conclude that the E344Q substitution in KPNA7, which was discovered in two patients with childhood epilepsy^23^, reduces binding of KPNA7 to both mono and bipartite NLS sequences.

### Identification of KPNA7 interacting proteins in neurons

Given that KPNA7 mutations are associated with epilepsy^23^, we set out to identify KPNA7-interacting proteins using extracts of neuronal origin. Previous studies have used tandem mass spectrometry (MS/MS) to identify KPNA7-interacting proteins in HEK293^34^ and pancreatic cancer cell lines^35^. Given the E344Q KPNA7 mutant shows a reduction in NLS binding, identification of KPNA7 cargo proteins could generate hypotheses for how mutation of KPNA7 affects neuronal differentiation. For our experiments, we utilized human induced pluripotent stem cell (iPSC)-derived neurons, stable isotope labeling by amino acids in cell culture (SILAC), and mass spectrometry (Fig. 3a). Briefly, iPSC-derived neurons were differentiated in neuron media for 2 weeks and then transferred to SILAC neuron media containing light and heavy isotopes of lysine and arginine (^13^C_6_,^15^N_2_-lysine/^13^C_6_,^15^N_4_-arginine (Lys8/Arg10)) for an additional two weeks. Four-week-old neurons were harvested, nuclear fractions prepared, and the light- and heavy-labeled nuclear lysates used for pull-down with MBP-beads and MBP-KPNA7-beads, respectively (Fig. 3a). The two bead types were then combined, processed for analysis by liquid chromatography and tandem mass spectrometry (LC-MS/MS) (Fig. 3a). In total, 1257 proteins were identified and abundance ratios (Heavy/Light) were calculated to examine protein enrichment in the MBP-KPNA7 pulldown (Supplementary Table 1). There were 40 proteins with abundance ratios greater than 2.75 and >10 identified peptides (Supplementary Table 2) which are shown in red in the abundance ratio plot (Fig. 3b). When analyzed for GO biological processes there was significant enrichment for proteins involved in processes related to RNA and mRNA regulation and splicing (Fig. 3c). Previous studies investigating KPNA7 interacting proteins by MS using HEK293 and pancreatic cancer cell lines also reported enrichment for proteins involved in RNA processing^34,35^.

### Characterization of hnRNP R and hnRNP U binding to KPNA7

Two heterogeneous nuclear ribonuclear proteins, hnRNP R and hnRNP U, showed large numbers of peptides (92 and 442 respectively) and a high abundance ratio (100 and 3.159 respectively), indicative of specific binding to KPNA7. hnRNP R and hnRNP U are part of the hnRNP family of ~20 RNA binding proteins that assemble into ribonucleoprotein complexes which function in RNA processing and transport^36,37^. Other hnRNP proteins were detected in our data, but because they had abundance ratios <2.75, these proteins were not pursued. hnRNP R and hnRNP U were initially identified as part of a large supramolecular heterogeneous ribonucleoprotein particle, but have also been shown to be associated with other smaller ribonucleoprotein complexes with functions in the nucleus and cytoplasm^38–41^. To determine if hnRNP R and hnRNP U are part of the same protein complex, we performed immunoprecipitation and immunoblotting with antibodies to the two proteins with extract from HEK293T cells. Immunoprecipitation of hnRNP U (120 kDa) resulted in virtually no recovery of hnRNP R (75 kDa) (Fig. 3d). This suggests the enrichment of hnRNP proteins on KPNA7 is via binding of the individual proteins or separate protein complexes. Gel filtration chromatography (Superose6) using these extracts revealed dissimilar elution profile weights for hnRNP R (~1 MDa) and hnRNP U (200–400 kDa) (Supplemental Fig. 1). These data suggest that at least two types of hnRNP particles, which contain hnRNP R and hnRNP U, can each bind to KPNA7. We also validated the interactions between KPNA7 and hnRNP R and hnRNP U using lysates from iPSC-derived neurons. MBP-KPNA7 and the mutant versions P339A and E344Q were immobilized on beads, combined with neuronal extracts, and the bound fractions analyzed by immunoblotting. We observed specific binding of hnRNP R and hnRNP U to WT KPNA7, and to the KPNA7 mutant P339A. Significantly, hnRNP R and hnRNP U binding to the KPNA7 mutant E344Q was dramatically reduced (Fig. 3e). Our data indicate that KPNA7 can bind to hnRNP proteins and that the E344Q mutation within ARM7 reduces binding.

### hnRNP R contains a bipartite NLS

**Figure 4:**
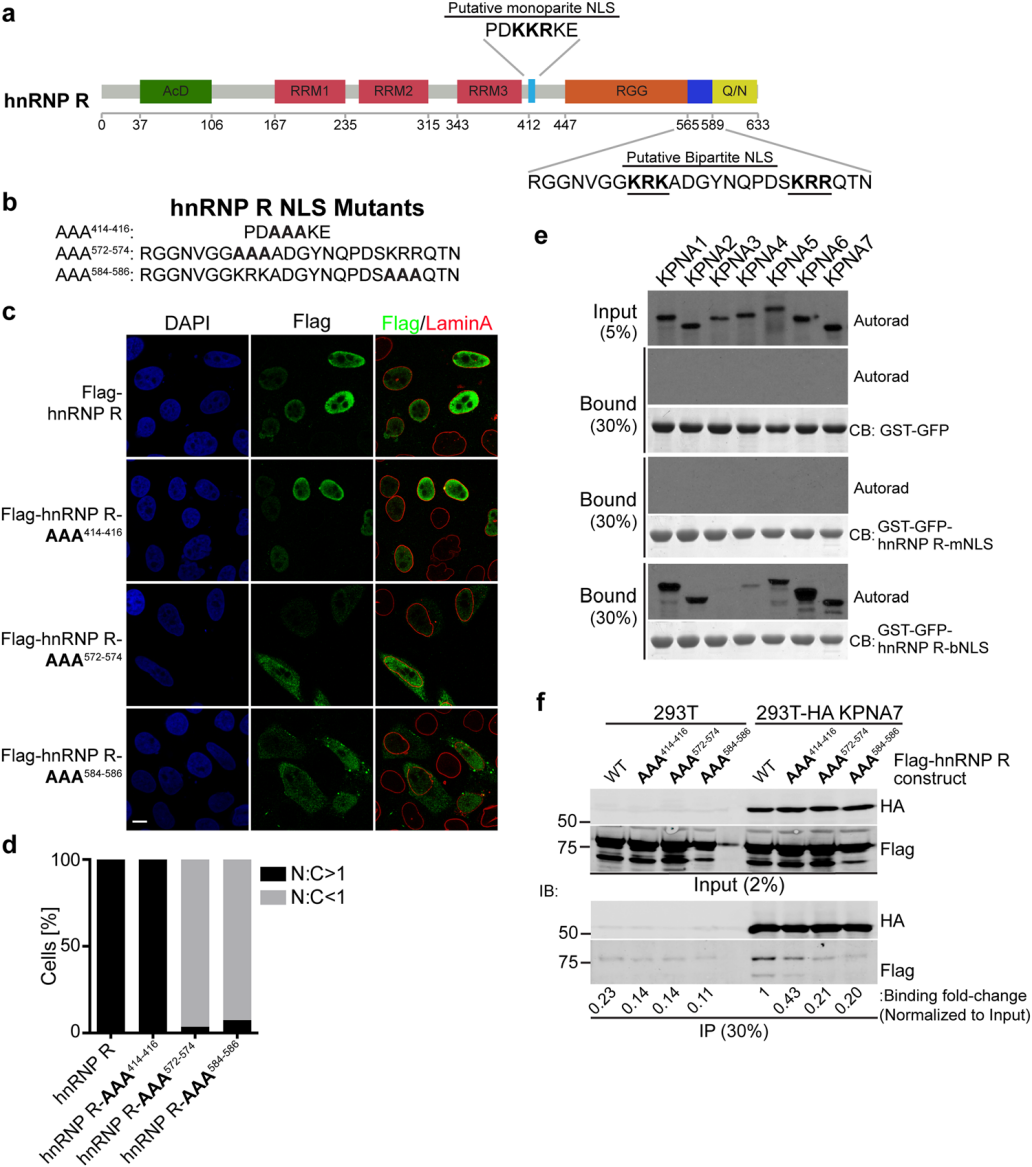
hnRNP R contains a functional bipartite NLS. (**a**) Diagram depicting hnRNP R domains. The domains are labeled as follows: AcD – Acidic Domain, RRM – RNA recognition motif, RGG – RNA-binding Arg-Gly-Gly box, Q/N – Glu and Asn rich domain. Putative mono and bipartite NLS are indicated. (**b**) Mutant constructs were made with the indicated residues substituted with Alanine. (**c**) Flag epitope-tagged NLS mutant constructs were expressed in HeLa cells and imaged by confocal immunofluorescence microscopy. Nuclear and cytoplasmic Flag intensity was quantified and the percentage of cells with nuclear-to-cytoplasmic (N:C) ratios of greater than or less than 1 is plotted in (**d**). (**e**) Putative monopartite and bipartite NLS were expressed as GST-GFP fusion proteins and immobilized on glutathione beads for use in a binding assay with ^35^S-labeled KPNA proteins. Bound fractions were analyzed by SDS-PAGE and autoradiography. (**f**) HA-immunoprecipitation was performed in HEK293T and HEK293T(HA-KPNA7) cell extracts after transfection with the indicated hnRNP R constructs, and analyzed by SDS-PAGE and western blot. Binding was quantified by Flag antibody blotting signal and normalized to input. Binding (fold-change) relative to WT hnRNP R binding to HA-KPNA7 is shown below each lane. Blots, films and gels in e and f were cropped for presentation; full images are in Supplementary Materials.

hnRNP R is a multi-domain protein that includes three RNA-recognition motifs and an RNA binding RGG (Arg-Gly-Gly) box (Fig. 4a). Based on protein sequence analysis and its similarity to the closely related hnRNP Q, hnRNP R was proposed to contain both a monopartite NLS (mNLS, a.a. 412-418) and a bipartite NLS (bNLS, a.a. 565-589) (Fig. 4a)^42,43^. The necessity and sufficiency of these NLSs in hnRNP R, however, have not been tested. To this end, we generated mutations within the putative mono- and bipartite NLSs that target basic residues that typically reduce NLS function (Fig. 4a,b amino acid changes in bold). Each mutant construct was introduced by transient transfection into HeLa cells and the subcellular localization determined by immunofluorescence confocal microscopy (Fig. 4c). We quantified the ratio of nuclear-to-cytoplasmic (N:C) localization of each construct and found the monopartite NLS mutant, AAA^414−416^, displayed a nuclear localization similar to the WT NLS (N:C ratio > 1) in 100% of cells (Fig. 4d). In contrast, amino acid substitutions in either element of the candidate bipartite NLS, AAA^572−574^ and AAA^584−586^ reduced nuclear localization in ~90% of the cells and showed an N:C ratios of less than 1. This result indicates that the bipartite NLS in hnRNP R (inclusive of the two basic elements identified) is responsible for nuclear localization of hnRNP R. We then evaluated the ability of the NLSs to bind import receptors. Each was expressed as a GST-GFP fusion protein, immobilized on beads, and used in pull-down binding assays with KPNA proteins labeled by *in vitro* translation with ^35^S-methionine (Fig. 4e). None of the isoforms bound to the proposed monopartite NLS, but multiple KPNA isoforms including KPNA7 bound to the bipartite NLS in hnRNP R (Fig. 4e). These binding data together with the localization data suggest the bipartite NLS of hnRNP R is used for import, potentially by multiple receptors including KPNA7.

### Transfected KPNA7 binds to the hnRNP R bipartite NLS

We next evaluated the effect of the hnRNP R NLS mutations on the interaction with KPNA7 in cells. We expressed the mutant hnRNP R constructs in a HEK293T cell line that stably expresses HA-KPNA7 and performed immunoprecipitation for the HA epitope (Fig. 4f). We observed co-immunoprecipitation of WT hnRNP R with KPNA7. After normalization to protein input levels, the two bipartite NLS mutants, AAA^572−574^ and AAA^584−586^, reduced binding to ~20% of the wild type protein, similar to background binding observed in control cells that lack HA-KPNA7. The hnRNP R AAA^414−416^ monopartite NLS mutant showed reduced binding to KPNA7. While we did not see an effect of this NLS on protein localization, nor binding to the isolated NLS sequence, we cannot rule out a contribution of this sequence on KPNA7 binding to full-length hnRNP R. Overall, our results indicate that the interaction between KPNA7 and hnRNP R is primarily through the bipartite NLS sequence, which is also necessary for nuclear localization in cells.

### KPNA7 binding to the bipartite NLS sequence in hnRNP R is reduced by the E344Q substitution

**Figure 5:**
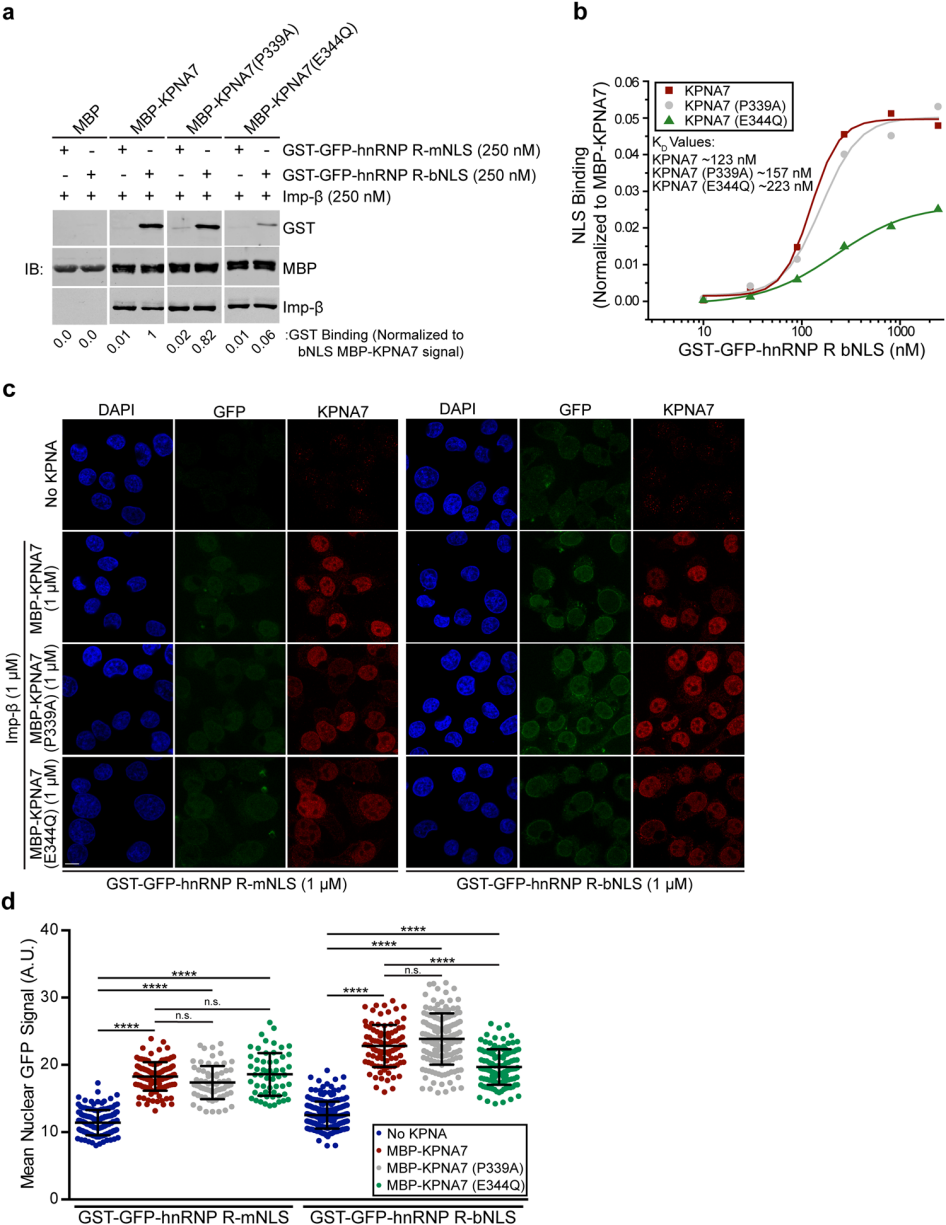
KPNA7 binding to the bipartite NLS of hnRNP R is reduced by the E344Q substitution. (**a**) Binding assay with the indicated MBP proteins bound to amylose resin and the indicated GST-GFP hnRNP R NLS constructs (250 nM) in the presence of Imp-*β* (250 nM). NLS binding was normalized to MBP signal and plotted below each lane. Blots were cropped for presentation; full-length blots available in Supplementary Materials. (**b**) Binding curve of GST-GFP-hnRNP R bipartite NLS to KPNA7 proteins generated by bead binding assay. Approximate K_*D*_values were generated and are shown. (**c**) Fluorescence microscopy images of permeabilized cell import assays with GST-GFP-hnRNP R NLS constructs. The assays were performed in HeLa cells permeabilized with 0.005% digitonin, and incubated with Ran (0.2 *μ*M), NTF2 (2 *μ*M) and an energy regenerating system with the recombinant factors indicated. Confocal IF microscopy was used to visualize. (**d**) Quantification of NLS import for each reaction. At least 100 cells of each were quantified. (Scale: 10 *μ*m. ****p < 0.0001).

We next tested how the epilepsy-associated mutations in KPNA7 affect binding to the hnRNP R NLS. We utilized recombinant MBP-KPNA7 fusion proteins for binding assays with GST-GFP-hnRNP R-NLS constructs (Fig. 5a). Robust binding of MBP-KPNA7 and MBP-KPNA7(P339A) to the hnRNP R bipartite NLS was observed, while MBP-KPNA7(E344Q) shows less binding (~10-fold less compared to WT KPNA7). The hnRNP R mNLS binding to KPNA7 was only slightly above background levels of binding to MBP (Fig. 5a). We generated apparent K_*D*_values for KPNA7, KPNA7(P339A) and KPNA7(E344Q) by performing binding assays with a range of GST-GFP-hnRNP R-bNLS concentrations (10 nM to 2.7 *μ*M) (Fig. 5b). We determined that KPNA7 binds to the hnRNP R-bNLS with an affinity of ~123 nM, while KPNA7(E344Q) shows nearly 2-fold lower binding with an affinity of ~223 nM.

To determine if KPNA7 can facilitate nuclear import of the two hnRNP R NLS sequences we performed digitonin-permeabilized cell import assays (Fig. 5c). We observed that KPNA7 addition resulted in an increase in nuclear GFP signal for the bipartite NLS reporter protein, and surprisingly, for the monopartite NLS as well (Fig. 5d). There was no significant difference in the nuclear accumulation of hnRNP R-mNLS between each of the KPNA7 constructs. The mutant KPNA7(E344Q) supported a lower level of nuclear import of hnRNP R-bNLS as compared to WT KPNA7 (15% reduction, p < 0.0001). Our data indicate that KPNA7 binds to, and facilitates nuclear import of, the hnRNP R bipartite NLS and that the E344Q substitution reduces this function.

### KPNA7 binding to the monopartite NLS sequence in hnRNP U is reduced by the E344Q substitution

**Figure 6:**
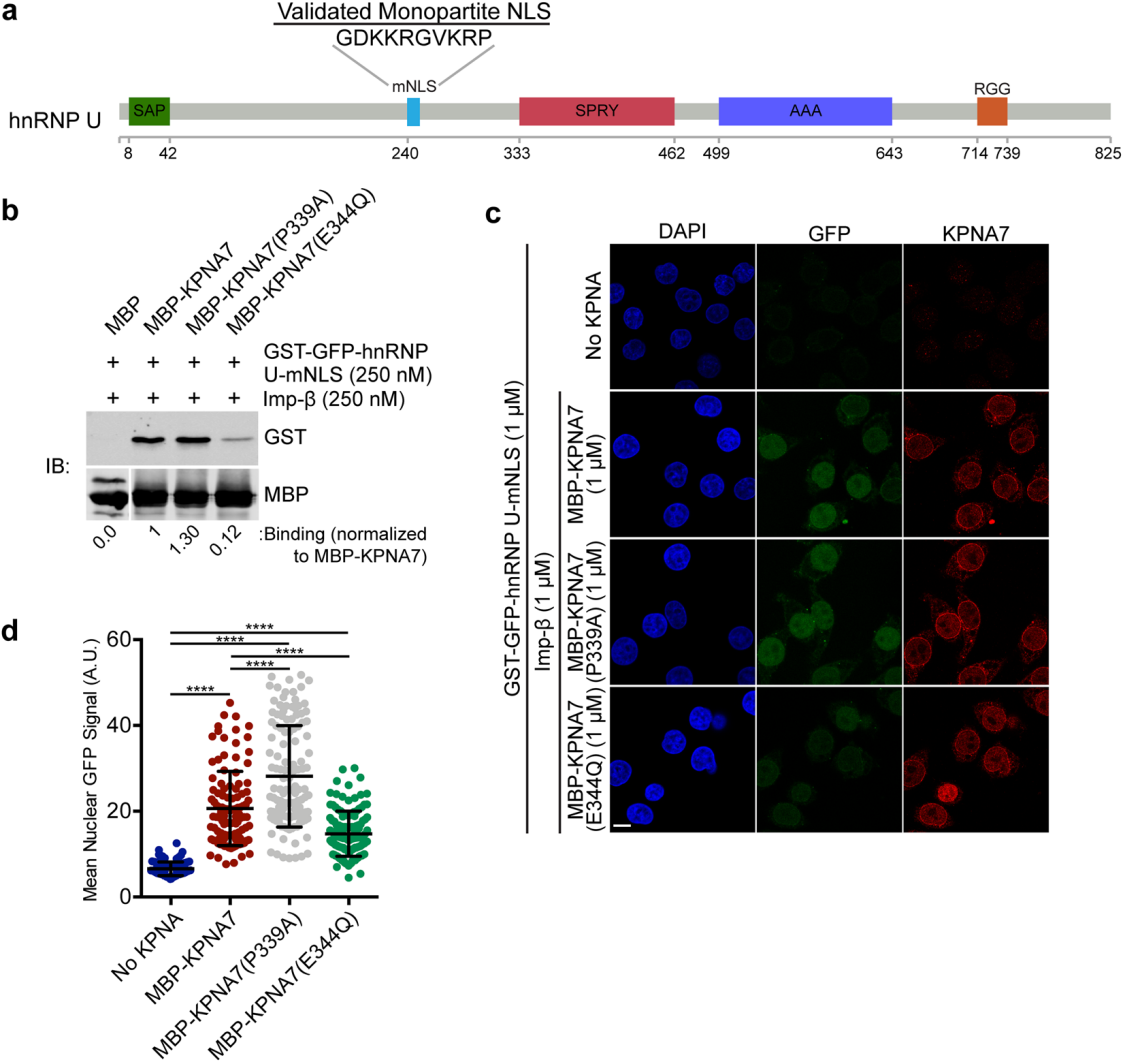
KPNA7 binding to the monopartite NLS of hnRNP U is reduced by the E344Q substitution. (**a**) Diagram depicting the domains in the hnRNP U protein. Domains shown are as follows: SAP – SAP DNA binding motif; mNLS – monopartite NLS sequence; SPRY – SPRY domain unknown function; AAA – ATPase domain; RGG – RNA-binding Arg-Gly-Gly box. (**b**) The monopartite NLS sequence from hnRNPU was expressed as a GST-GFP fusion protein and used for a binding assay with MBP-KPNA7 proteins on amylose beads in the presence of Imp-*β* (250 nM). Normalized binding was calculated and is shown below each lane. Blots were cropped for presentation; full-length blots available in Supplementary Materials. (**c**) Fluorescence microscopy images of permeabilized cell import assays with GST-GFP-hnRNP U NLS. The assays were performed in HeLa cells permeabilized with 0.005% digitonin, and incubated with Ran (0.2 *μ*M), NTF2 (2 *μ*M) and an energy regenerating system with the recombinant factors indicated. (**d**) Quantification of NLS import for each reaction. At least 100 cells of each were quantified. (Scale: 10 *μ*m; ****p < 0.0001).

hnRNP U is structurally distinct from hnRNP R and unique among the hnRNP family in that it does not contain an RNA recognition motif^44^. Rather, hnRNP U binds RNAs through the arginine and glycine-rich RGG box at the C-terminus (Fig. 6a)^44^. Interestingly, while a monopartite NLS sequence was identified in hnRNP U and the protein is strongly nuclear in localization, early heterokaryon studies determined that hnRNP U is confined to the nucleus and does not undergo nucleocytoplasmic shuttling in HeLa cells^44–46^. Regardless, the nuclear localization of the protein was shown to be dependent on the NLS sequence46. We expressed the monopartite NLS sequence from hnRNP U as a GST-GFP fusion for binding and transport assays. All three KPNA7 proteins tested bound the hnRNP U-mNLS sequence. In the presence of Imp-*β*, though, binding to the KPNA7(E344Q) mutant was ~8-fold less than binding to WT or P339A forms of KPNA7 (Fig. 6b). Similar levels of binding were observed between WT KPNA7 and the P339A mutant.

To determine if KPNA7 can facilitate import of the hnRNP U NLS sequence, we performed digitonin-permeabilized cell import assays (Fig. 6c). Significant, KPNA7-dependent nuclear accumulation of the GST-GFP-hnRNP U-mNLS sequence was observed (Fig. 6d). Consistent with our observations for other NLS sequences, KPNA7(E344Q)-dependent import was significantly reduced (~30% reduction, p < 0.0001) compared to WT KPNA7. These data further demonstrate that the E344Q substitution in KPNA7 reduces the transport function of the receptor for both monopartite and bipartite NLS sequences.

### KPNA7 expression is induced during neurogenesis

**Figure 7:**
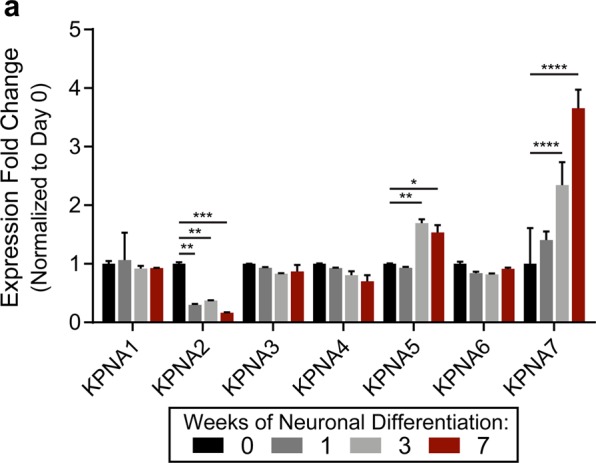
KPNA expression levels during human neuronal differentiation. (**a**) RT-qPCR analysis of the human KPNA1-7 genes during the neuronal differentiation of iPSC-derived neural progenitor cells (NPCs). Expression is normalized to the NPCs at 0 weeks of differentiation for each isoform. Significant differences in expression are indicated. (****p < 0.0001, ***P < 0.001, **p < 0.01, *p < 0.05).

The effect of mutations in KPNA7 on neuronal development suggests a possible function for KPNA7 during neurogenesis^23^. Moreover, expression changes of certain KPNA proteins (isoform switching^18^) have been linked to neural development. We used human induced pluripotent stem cell (iPSC)-derived neural progenitor cells (NPCs) as a model to examine the expression profile of KPNA7 during neurogenesis. In brief, iPSC-derived NPC cultures were treated with neurogenic factors to induce differentiation for seven weeks, and qPCR was used to query the mRNA levels of KPNA isoforms. We used GAPDH levels to normalize all KPNA expression values, and the data were plotted as the fold-change relative to NPCs (Day 0 of differentiation). We observed a significant increase (~2.3-fold) in *KPNA7* expression from NPC (0 weeks differentiation) to three weeks (Fig. 7a). After seven weeks of differentiation, KPNA expression increased ~3.6-fold relative to NPCs (Fig. 7a). It should be noted however that at seven weeks of differentiation *KPNA7* transcript levels are still at least ~15-fold lower than the other Imp-*α* isoforms. Regarding the other isoforms, *KPNA2* is highly expressed in NPCs but its expression is reduced significantly (~6.2-fold) during differentiation. Additionally, there is a small increase (~0.7-fold) in the expression of *KPNA5* by three weeks of differentiation. There are negligible changes in expression for the other Imp-*α* isoforms. From these data, we conclude that there are significant changes in expression of select Imp-*α* isoforms during neuronal differentiation, and this includes induction of *KPNA7* expression.

### Exon 7 of KPNA7 contains a CTCF binding motif

**Figure 8:**
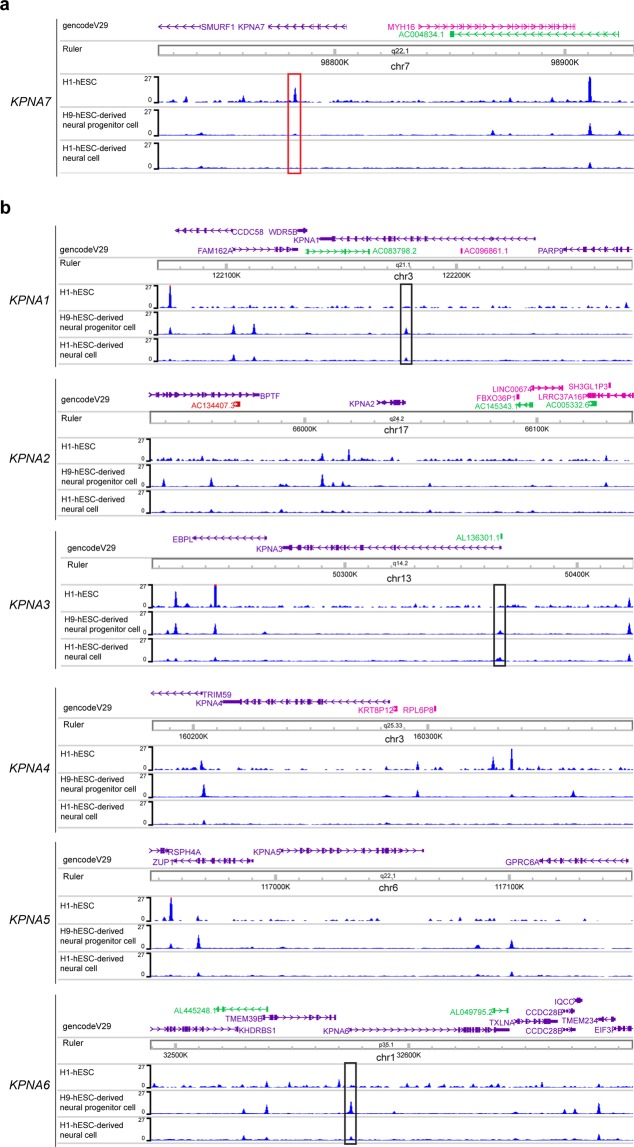
CTCF occupancy of *KPNA7* exon 7 is lost during neuronal differentiation of stem cells. (**a**) CTCF ChIP-Seq profiles from three ENCODE datasets (ENCFF619IWL, ENCFF940XMP, ENCFF259PXQ) of H1-ESCs, H9-ESC-derived neural progenitor cell, and an H1-ESC-derived neural cell at the *KPNA7* gene locus. A red box indicates the CTCF peak on Exon 7 of *KPNA7* which is lost during differentiation. (**b**) CTCF ChIP-Seq profiles from the same datasets for the other six Imp-*α* genes. Black boxes are shown around peaks in each gene which change with differentiation.

The mechanism that drives *KPNA7* expression changes during development has not been defined. We noted that within exon 7 of KPNA7, there is a sequence (c.1020–1038) with high similarity to the consensus site for the transcription factor CCCTC-binding factor (CTCF). CTCF is a well-described transcriptional insulator composed of 11 zinc-finger DNA-binding domains^47^. About 5% of CTCF binding sites in the genome are exonic, where it can mediate transcription inhibition^48^ and regulation of alternative splicing^49^. We examined if CTCF is bound to exon 7 of *KPNA7* in stem cells, neural progenitors cells and neurons by using CTCF ChIP-Seq data from the Encode Project (ENCFF619IWL, ENCFF940XMP, ENCFF259PXQ) (Fig. 8a). In hESCs, there is CTCF signal at exon 7 of *KPNA7* (red box); the CTCF peak signal in exon 7 is greatly reduced in hESC-derived neural progenitor cells and essentially absent from the hESC-derived neural cell. The reduction in CTCF binding to exon 7 of KPNA7 during neuronal differentiation is occurring within the time frame that total CTCF expression is known to decrease^50^. We examined if CTCF occupancy is correlated with the expression of other KPNA isoforms during neurogenesis in the same CTCF Chip-Seq dataset (Fig. 8b). We observed several changes in peaks of CTCF binding intronic to *KPNA1*, *KPNA3*, and *KPNA6* (black boxes), however, none of these peaks show the same differentiation-associated reduction in binding that was detected in exon 7 of *KPNA7*.

**Figure 9:**
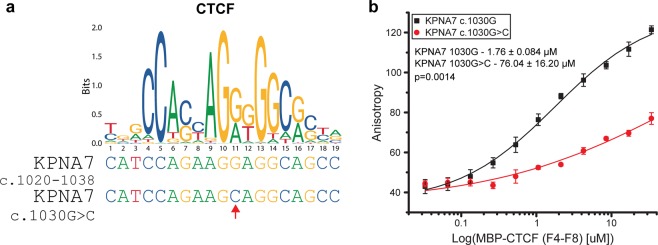
The 1030G > C transversion significantly reduces CTCF binding affinity. (**a**) CTCF binding motif sequence logo with the *KPNA7* sequences shown underneath. The c.1030G > C transversion is marked with a red arrow. (**b**) Fluorescence polarization anisotropy analysis of recombinant MBP-CTCF (Zinc Fingers 4-8) binding to fluorescently labeled dsDNA oligos containing the 16 base-pair *KPNA7* CTCF binding motif. The G > C transversion reduces CTCF affinity from 1.76 ± 0.084 *μ*M to 76.04 ± 16.2 *μ*M.

**Figure 10:**
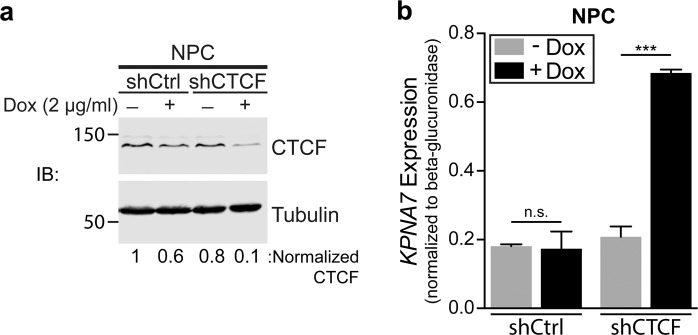
CTCF knockdown in neural progenitor cells increases *KPNA7* expression. (**a**) Western blot analysis of CTCF protein levels in shCTCF and shCtrl NPCs with and without doxycycline (dox) treatment for 7 days. CTCF protein levels were normalized to tubulin and then relative to the control condition. Blots in d and e were cropped for clarity, full-length blots available in Supplementary Materials. (**b**) RT-qPCR expression analysis of *KPNA7* transcript levels in shCTCF and shCtrl NPCs. Expression was normalized to beta-glucuronidase. Significance is shown. (****p < 0.0001).

The CTCF binding motif in exon 7 (c.1020-1038) of *KPNA7* overlaps the epilepsy-associated c.1030G > C transversion (Fig. 9a). Since this is the transversion that results in the E344Q substitution, it raised the possibility that this mutation might affect both KPNA7 NLS binding and KPNA7 expression. To evaluate the effect of the exon 7 mutation on CTCF binding, we performed polarization anisotropy measurements with recombinant CTCF (Zinc fingers 4–8) and fluorescently labeled dsDNA oligos that comprise the WT and mutated KPNA7 exon 7 DNA sequences (Fig. 9b). CTCF bound to the WT DNA sequence with an apparent K_*D*_ = 1.76 ± 0.084 *μ*M, but CTCF binding to the mutated DNA sequence was significantly lower, with an apparent K_*D*_ = 76.04 ± 16.20 *μ*M) (p = 0.0014). Our data predict that CTCF binding to exon 7 of KPNA7 would be significantly reduced by the c.1030 G > C transversion detected by WES in the patients. To test whether CTCF regulates KPNA7 expression in NPCs, we generated stable NPC lines with doxycycline (dox)-regulated shRNAs targeting CTCF, and a scrambled shRNA control (Fig. 10a,b). We determined that dox-induction of CTCF shRNA resulted in decreased CTCF expression and increased KPNA7 expression (~3.3-fold, p < 0.001; Fig. 10a,b). This result suggests that CTCF plays a role in regulating *KPNA7* expression in NPCs. These data, combined with the ChIP-Seq analysis, suggest that CTCF binding to exon 7 of *KPNA7* is part of a repressive mechanism that maintains a low level of KPNA7 until neurogenesis.

## Discussion

The KPNA family of transport receptors share 40–85% identity and have a highly conserved structure that consists of an auto-inhibitory N-terminal IBB domain and a core structure based on ARM repeats^9,11^. Each ARM repeat contains ~40 amino acids that form a three-helix structure^51^. The 10 ARM repeats are stacked in a manner that creates a concave surface used for NLS binding^11^. The interaction sites for transport signals include a major NLS binding groove between ARMs 2–4 and a minor NLS binding groove between ARMs 6–8. The major groove is responsible for interactions with both monopartite and bipartite NLS sequences, while the minor groove is primarily used in binding to bipartite NLS sequences. Despite the highly conserved structure and function of KPNA family members, the expression profiles of KPNA receptors suggests there is a high degree of specialization for particular KPNA receptors in defined cellular contexts. KPNA7 expression is low in most adult cell types but has been shown to have essential roles in embryo development in mice^29^. Kpna7 knockout mice displayed reduced reproductive function and a sex imbalance with preferential lethality in females, in both heterozygous and homozygous Kpna7-mutant embryos^29^. Similar roles for KPNA7 have been observed in the study of porcine, bovine, and trout embryos and cell lines^28,30,31^. These data, together with the neurodevelopmental phenotypes in the two epilepsy patients, suggest that KPNA7 activity is important in a variety of developmental contexts^23^.

We set out to determine how epilepsy-associated mutations in KPNA7 affect its activity as a nuclear import receptor^9,23^. Each of the mutations gives rise to amino acid substitutions that, by protein modeling, are proximal to the minor NLS binding grove. This inference is based on threading KPNA7 onto the crystal structure of KPNA2^23^. Because the affected siblings inherited one mutant allele from each of the parents, who were asymptomatic, the epilepsy phenotypes were proposed to occur as a consequence of compound heterozygosity^23^. Thus, patient cells would be predicted to express two forms of KPNA7, one containing the E344Q substitution and another containing P339A. We engineered these amino acid substitutions into KPNA7 and determined that the E344Q substitution results in a reduction in NLS binding. The E344Q substitution results in loss of a negatively charged amino acid from a position that is conserved in all seven KPNA isoforms. The impact of the E344Q substitution on bipartite NLS binding can be rationalized in terms of reduced function of the minor NLS binding site. Why the E344Q substitution affects monopartite NLS binding is unclear. One possibility is that the E344Q substitution induces a structural change that is transduced to the major NLS binding site. It is also possible that the minor NLS binding site in KPNA7 can be used for monopartite NLS binding, an idea suggested by KPNA2 co-crystalized with NLS peptides^11,52^. Our assays did not detect an effect of the P339A substitution on NLS binding to KPNA7. This suggests the contribution of this substitution to the disease state is not directly related to cargo engagement, however it remains possible there is an effect that is manifest with cargoes that were not included in our analysis. Neither the P339A nor the E344Q substitution had a significant effect on Imp-*β* binding, suggesting the accessibility of the N-terminal IBB is comparable in the WT and mutant forms of KPNA7.

Several studies have identified NLS-containing proteins that bind KPNA7, and in a few cases, demonstrated these are cargoes for KPNA7-dependent transport^34,35^. Reasoning that epilepsy reflects changes in neuronal activity, we used SILAC labeling and a straightforward MS/MS pipeline to identify KPNA7-interacting proteins in hiPSC-derived neurons. Our analysis found a significant enrichment of proteins involved in RNA and mRNA processing, including hnRNP R and hnRNP U. We validated the biochemical interactions of KPNA7 with hnRNP R and hnRNP U, and we also determined the importance of putative mono- and bipartite NLS sequences in these hnRNPs^42,43^. We found that the E344Q substitution in KPNA7 reduced binding and transport mediated by the NLS in hnRNP R and hnRNP U. Our findings raise the interesting possibility that the E344Q substitution might compromise hnRNP import in the setting of neurons. This model is speculative, however, and it is complicated by the fact that multiple import receptors can bind and transport hnRNP proteins. It is interesting that both hnRNP R and hnRNP U have been linked to neuronal function and development. hnRNP R associates with the survival motor neuron protein (SMN) in axons to promote neurite outgrowth in neuronal differentiation^53,54^. Mutations in hnRNP R, including an amino acid substitution in the bipartite NLS, have been associated with abnormalities of the brain, involving severe developmental delay, seizures, and corpus callosum and cerebellar abnormalities^55^. Variants in hnRNP U have been linked to neuronal development and disorders such as encephalopathies, intellectual disabilities, and epilepsy^56–59^. These studies suggest that reduced import of hnRNP R and hnRNP U could result in severe neurodevelopmental defects similar to those observed in the patients with mutations in KPNA7.

Modulation of nuclear import via differential expression of KPNA isoforms is critical for developmental programs^15,16,60,61^. A process termed Imp-*α* isoform (or subtype) switching discovered by the Yasuhara group was shown to be important for mouse neuronal differentiation^18^. In mice, differential expression of Kpna1 and Kpna2 results in distinct transport outcomes for pro-pluripotency and pro-neural NLS containing proteins including Oct3/4, Sox2, Oct6 and Brn2^18^. Increased expression of Kpna1 and decreased expression of Kpna2 helped promote differentiation toward a neuronal state, and Kpna4 expression was shown to increase during differentiation^18^. We detected evidence for KPNA isoform expression changes during the differentiation of human iPSC-derived NPCs to neurons. We noted a *KPNA2* reduction during differentiation in human cells, which is similar to what was observed in the mouse study, but we did not detect a significant change in *KPNA1* or *KPNA4*. Additionally, we observed increased expression of *KPNA5* and *KPNA7*, genes that were not examined in the mouse study. Our data imply that KPNA7 is one of the import receptors that undergoes expression changes, potentially in order to regulate transport of cargoes with functions that are important for differentiation.

Analysis of one of the mutant KPNA7 alleles also suggested a causal link between CTCF occupancy and KPNA7 expression. Exon 7 in the KPNA7 gene contains a CTCF binding site, and by ChIP-seq the occupancy of CTCF at this site is reduced during neuronal differentiation. Remarkably, the c.1030G > C transversion detected in the epilepsy patients is contained within the CTCF binding site. We used fluorescence anisotropy to show that CTCF binding to this site is reduced approximately ~40-fold by the c.1030G > C transversion. Moreover, neuronal-depletion of CTCF was sufficient to increase KPNA7 expression level. These data strongly suggest that CTCF contributes to the negative regulation of KPNA7 expression, and the mechanism can be partially relieved by the mutation. Other studies have shown that CTCF has important roles in neuronal development, and that there is an overall decrease in both CTCF expression and the number of CTCF ChIP-Seq peaks during neuronal differentiation^50,62,63^. We propose that a CTCF-dependent mechanism might be important for regulating KPNA7 expression during neuronal differentiation. It is plausible that the c.1030G > C transversion identified in patients with severe neurodevelopmental defects significantly reduces CTCF binding to the *KPNA7* gene, and that precocious expression of KPNA7 during development contributes to the disease phenotype.

## Methods

### Protein modeling

The crystal structure of KPNA7 has not been solved. To generate a model structure for KPNA7 we utilized the SWISS-MODEL software to thread the KPNA7 primary sequence onto the crystal structure from mouse KPNA2 (PDB: 1PJM). The epilepsy-associated amino acid substitutions were made in PyMol. Images were rendered in PyMol^64^.

### Plasmids

The human KPNA7 protein sequence was codon-optimized for *E. coli* (Genewiz) and cloned into pMBPHis-Parallel1^65^ for expression as a maltose binding protein fusion. PCR mutagenesis was performed using Pfu Ultra II (Agilent) to introduce the P339A and E344Q amino acid substitutions. The NLS sequences used in this study (SV40, DDB2, hnRNP R mNLS, hnRNP R bNLS, hnRNP U) were cloned into the pGEX-4T1-GFP vector. Flag-myc-hnRNP R plasmid (RC224502) was obtained from OriGene. Mutagenesis PCR was performed using Pfu Ultra II (Agilent) to generate the NLS mutant constructs. The CTCF expression construct^66^ is in the pMAL-c2g backbone, which contains Zinc fingers 4–8 of human CTCF, and was a gift from Gary Felsenfeld (NIH).

### Recombinant protein expression

Standard methods were used to express MBP-KPNA7 fusion proteins in BL21 *E. coli* at 18 °C overnight. Cultures were harvested, lysed by French Press, clarified and incubated with amylose resin (NEB). After extensive washing, MBP-fusion proteins were eluted with 10 mM maltose and dialyzed into 50 mM Tris pH 8.0, 200 mM NaCl with 1 mM DTT. Standard methods were also used to express GST constructs in BL21 *E. coli* at 18 °C overnight. *E. coli* pellets for GST fusions were processed in a similar manner to the MBP fusions, except they were incubated with glutathione resin (Millipore-Sigma). GST-GFP-NLS constructs were dialyzed into PBS with 1 mM DTT. Plasmid encoding MCP-CTCF(F4-F8) was transformed into BL21 *E. coli* and expression was induced with 1 mM IPTG in the presence of 100 *μ*M ZnCl_2_ for 2.5 hours at 37 °C. Cultures were harvested, and lysed by French press in 50 mM Tris pH 8, 200 mM KCl, 100 *μ*M ZnCl_2_, and 1 mM DTT with protease inhibitors. During lysis, Pierce Universal Nuclease was included to digest nucleic acids. The lysate was incubated with amylose resin and washed extensively with 2 M KCl. MBP-CTCF(F4-F8) was eluted with 10 mM Maltose in 20 mM Tris pH 8, 200 mM KCl, 100 *μ*M ZnCl_2_, 1 mM EDTA, 1 mM DTT, and 1 *μ*g/ml each of leupeptin, pepstatin, and aprotinin. Purified protein was dialyzed into the same buffer to remove maltose. All recombinant proteins were analyzed by SDS-PAGE for purity and quantified by Bradford protein assay. Methods for expression and purification of Imp-*β*, Ran, NTF2, and CAS have been published^9,67,68^.

### Binding assays

MBP fusion proteins were bound to amylose resin (1 *μ*g protein/*μ*l resin) for >3 hrs at 4 °C and then blocked for at least 1 hr with 1 mg/ml BSA. Imp-*β* and GST-GFP-NLS constructs were incubated with 10 *μ*l beads (resin indicated) at the stated concentrations in a total reaction volume of 100 *μ*l for 2 hrs at 4 °C in amylose column buffer (20 mM Tris pH 8, 200 mM NaCl, 1 mM EDTA, 1 mM DTT, 0.05% Triton X-100 and 1 *μ*g/ml each of leupeptin, pepstatin, and aprotinin. After binding, beads were washed five times with 400 *μ*l of the same buffer and analyzed by SDS-PAGE and western blot. Blot quantification was performed with ImageStudio (Li-Cor).

For the radiolabeled binding assay, the Imp-*α* isoforms were expressed as ^35^S-methionine-labeled proteins by *in vitro* transcription/translation (TnT coupled reticulocyte lysate system; Promega). Scintillation counting was used to measure incorporation and normalize inputs. GST-fusion proteins were bound to glutathione resin (1 *μ*g/*μ*l resin). Reactions with a total volume of 100 *μ*l contained 10 *μ*l of beads. Each radiolabeled Imp-*α* protein (125,000 cpm) was added to reactions assembled in 25 mM Tris pH 7.5, 50 mM NaCl, 5 mM MgCl2, 0.1 mM EDTA, 0.5 mg/ml BSA, 0.1% NP-40, and 1 mM DTT with leupeptin, pepstatin, and aprotinin (1 *μ*g/ml each). Binding reactions were incubated for 3 hours at 4 °C. After binding, beads were washed five times in the same buffer, analyzed by SDS-PAGE and exposed to X-ray film.

### Cell culture

Neural progenitor cells derived from 9429 and BJ human fibroblasts (Coriell Cell Repository) were reprogrammed into induced pluripotent stem cells (iPSC) and induced to neural progenitor cells as previously described^69,70^. NPC’s were grown on Matrigel (Corning) in NPC medium, (DMEM/F12 + Glutamax (Invitrogen), 1X N2 (Invitrogen), 1X B27-Vitamin A (Invitrogen), 1ug/ml Laminin (Invitrogen), 20 ng/ml FGF-2 (Peprotech)), and passaged 1:6 every 4 days using Accutase (Innovative Cell Technologies). iPSC-derived neurons were cultured in neuron medium (DMEM/F12 + Glutamax, 1X N2, 1X B27 with Vitamin A (Invitrogen), 20 ng/ml BDNF (Shenandoah Biotechnology), 20 ng/ml GDNF (Shenandoah Biotechnology), 1 mM dibutyryl-cyclic AMP (Sigma), and 200 nM ascorbic acid (Stem Cell Technologies). Low passage (p4) BJ NPCs were used to generate doxycycline-inducible shRNA lines with control or CTCF targeted shRNAs. Transduced cells were selected by puromycin selection (0.4 *μ*g/ml) for 72 hours and maintained in 0.25 *μ*g/ml puromycin. For knockdown experiments, shRNA expression was induced with 2 *μ*g/ml doxycycline for 7 days.

Adherent HeLa cells (ATCC) were grown in DMEM-High Glucose (Gibco) with 10% FBS (Atlanta Biologics). HeLa cells were transfected with ViaFect reagent (Promega). HEK293T cells (ATCC) were cultured in in DMEM (Gibco) with 5% FBS (Atlanta Biologics), 1% non-essential amino acids (Gibco), 1% sodium pyruvate (Gibco) and 1% Pen/Strep (Gibco). The HA-KPNA7 HEK293T cell line was generated by lentiviral transduction. Virus was produced by Fugene6 (Promega) mediated transfection of HEK293T cells with pWPI-HA-KPNA7, and the packaging plasmids psPAX2 (Addgene #12260) and pMD2.G (Addgene #12259). Viral supernatant was filtered (0.45 *μ*m) prior to transduction. GFP-positive clonal lines were generated using the CellRaft AIR System (Cell Microsystems).

### Permeabilized cell import assays

Digitonin-permeabilized cell import assays were performed as previously described^71^. HeLa cells were plated on coverslips at a density of 0.15 × 10^6^ cells per ml 24 hours before each assay. Cells were washed 3x with ice-cold transport buffer (20 mM HEPES [pH 7.4], 110 mM potassium acetate, 2 mM magnesium acetate, 0.5 mM EGTA) containing 1 mM dithiothreitol (DTT) and 1 *μ*g/ml portions (each) of leupeptin, pepstatin, and aprotinin and then permeabilized with 0.005% digitonin for 5 min on ice. Import reactions contained an energy-regenerating system (5 mg/ml of bovine serum albumin, 80 U of creatine phosphokinase/ml, 1.6 mg of creatine phosphate/ml, 1 mM ATP, 1 mM GTP), 1 *μ*M NTF2 and 2 *μ*M Ran, as well as 1 *μ*M import substrate (GST-GFP fusion), 1 *μ*M Importin-*β* and/or 1 *μ*M MBP-KPNA7 as indicated. Reactions were carried out for 30 minutes at 30 °C and terminated by transferring coverslips to ice-cold transport buffer. After three washes in transport buffer, coverslips were processed for confocal immunofluorescence microscopy, as described below. Quantification of mean nuclear intensity was quantified using ImageJ software by creating a mask using the DAPI channel. One-way ANOVA with multiple comparisons was used for statistical analysis in GraphPad Prism.

### 2-Color bead-based fluorescence imaging assay

The bead-based fluorescence imaging assay used in this study was adapted from Rexach and colleagues^33^. Briefly, amylose resin was pre-loaded with protein (1 *μ*g/*μ*l packed beads) for at least 4 hours at 4 °C in 20 mM Tris pH 8, 200 mM NaCl, 1 mM EDTA, 1 mM DTT with leupeptin, pepstatin, and aprotinin (1 *μ*g/ml each) and blocked with 5 mg/ml BSA. MBP was labeled with IRDye-680 (LiCor) and added to control MBP beads at 0.05 *μ*g/*μ*l. MBP and MBP-KPNA7 beads were then combined at a ratio of 1:1 and made to a 50% slurry. Reactions were assembled in siliconized microcentrifuge tubes with 1.5 *μ*l bead slurry, 1 *μ*l 4X reaction buffer (40 mM EDTA, 40 mg/ml BSA, 500 mM NaCl), x *μ*l GST-GFP cargo (750 nM, final concentration), y *μ*l Imp-*β* (750 nM, final concentration), and distilled water to a total volume of 4 *μ*l. Reactions were then incubated for 10 minutes at room temperature, washed 2x with 200 *μ*l of 1X reaction buffer and transferred in 20 *μ*l total volume to a 96 well plate well for imaging. Beads were imaged by phase-contrast, and with a Cy5 and GFP filter using the EVOS Fl Imaging System (Invitrogen) with a 20x objective at equivalent intensity and exposures. The GFP and 680 signals were quantified using ImageJ and separated into 680 positive and negative (MBP and MBP-KPNA7 beads respectively) and GFP signal was plotted. GraphPad Prism was used for statistical analyses.

### KPNA7 cargo identification by SILAC based-mass spectrometry

NPCs derived from 9429 hiPSCs were plated onto Matrigel in T75 culture flasks. Upon reaching 80% confluency, neuronal differentiation was induced by switching to neuron media. Media was changed every 4 days for 2 weeks. Plates were gently washed 2x with PBS and switched to SILAC neuron medium. Basal SILAC neuron media contains DMEM/F12 for SILAC (ThermoFisher), which is deficient in Lysine and Arginine, 1X Glutamax (ThermoFisher), and the supplements in neuron media described above. Heavy SILAC media was made by supplementing the basal medium with 0.669 mM L-Arginine:HCl (^13^C_6_, ^15^N_4_) (Cambridge Isotope Laboratories) and 0.498 mM L-Lysine:HCl (^13^C_6_, ^15^N_2_) (Cambridge Isotope Laboratories). Light SILAC media was made by supplementing the basal medium with 0.669 mM L-Arginine:HCl (ThermoFisher) and 0.498 mM L-Lysine:HCl (ThermoFisher). Neurons were grown for 2 more weeks in each SILAC medium with media changes every three days for a total of 4 weeks of differentiation. Cells were harvested by scraping into cold PBS with 1 mM PMSF and pelleted by centrifugation. To check incorporation of the heavy amino acids, total cell lysate was analyzed by liquid chromatography-mass spectrometry (LC-MS). Greater than 1000 proteins were identified with an average heavy incorporation of ~85%.

Nuclear lysates were generated by incubating the neuronal pellets in 25 mM HEPES pH 7, 25 mM KCl, 0.05 mM EDTA, 5 mM MgCl_2_, 0.1% NP-40, 10% glycerol, and 1 mM DTT with leupeptin, pepstatin, and aprotinin (1 *μ*g/ml each) and incubating with shaking at 4 °C for 15 minutes. The insoluble nuclei were pelleted by centrifugation at 3K rpm for 5 minutes. Nuclear proteins were then released by 10-minute incubation in 50 mM HEPES pH 7.6, 400 mM KCl 0.1 mM EDTA and 1 mM DTT with leupeptin, pepstatin, and aprotinin (1 *μ*g/ml each). Lysates were then centrifuged for 20 minutes at 13K rpm and the soluble nuclear lysate was removed for use in pull-downs. Lysates were quantified by Bradford protein assay. Pull-downs were performed by immobilizing 40 *μ*g of MBP or MBP-KPNA7 on magnetic amylose resin (NEB). Beads were washed extensively with amylose column buffer. Equal amounts of the heavy and light lysates were loaded onto the MBP-KPNA7 and MBP beads respectively in the presence of 0.5 *μ*M his-Importin *β*. Reactions were incubated with agitation at 4 °C for 3 hours and then washed with amylose column buffer 5 times.

The light and heavy samples were combined in equal amounts for LC-MS/MS analysis performed by the W.M. Keck Biomedical Mass Spectrometry Laboratory at the University of Virginia as previously described^72^. Briefly, the sample was reduced with DTT (10 mM, 1 hr, RT) followed by alkylation with iodoacetamide (50 mM, 1 hr, RT). The sample was then digested overnight with alkylated trypsin (1 *μ*g; Promega, V5111). The digestion was quenched with 3 *μ*l acetic acid. The mixed solution was evaporated to 20 *μ*L for MS analysis. The LC-MS system consisted of a Thermo Electron Q Exactive HFX mass spectrometer system with an Easy Spray ion source connected to a Thermo 75 *μ*m × 15 cm C18 Easy Spray column. The sample was injected, and the peptides eluted from the column by an acetonitrile/0.1 M formic acid gradient at a flow rate of 0.3 *μ*L/min over 2.0 hours. The nanospray ion source was operated at 1.9 kV. The digest was analyzed using the rapid switching capability of the instrument acquiring a full scan mass spectrum to determine peptide molecular weights followed by product ion spectra (10 HCD) to determine amino acid sequence in sequential scans. This mode of analysis produces approximately 30000 MS/MS spectra of ions ranging in abundance over several orders of magnitude. The data were analyzed by database searching using the Sequest search algorithm against Uniprot human. The abundance ratios of all identified proteins were generated using all unique and non-unique peptides with ProteomeDiscoverer software. There were 41 proteins with greater than 10 identified peptides and an abundance ratio of greater than 2.75. PANTHER (pantherdb.org) was used to analyze these 41 genes for gene ontology biological process enrichment.

### Confocal immunofluorescence microscopy

Coverslips from transport and transfection assays were fixed with 3.75% formaldehyde for 15 minutes, permeabilized with 0.02% Triton-X for 5 minutes and blocked with 2% FBS and 2% BSA in PBS for 1 hour. Primary antibodies (anti-Flag M2 (mouse monoclonal, F1804, Sigma), anti-LaminA (rabbit polyclonal, L1293, Sigma), anti-KPNA7 (rabbit polyclonal, HPA031395, Sigma)) were diluted in blocking buffer and incubated at room temperature for 2 hours. Secondary antibodies (Goat Anti-Rabbit-Cy3 (115-165-144, Jackson ImmunoResearch), Donkey anti-mouse-FITC (715-225-151, Jackson ImmunoResearch)) were diluted in blocking buffer and incubated at room temperature for 1 hour. DAPI was used to stain nuclei. Images were acquired by laser scanning confocal microscopy (Zeiss 880 LSM, Carl Zeiss) at 40×, 1.3 NA oil immersion objective with Zen software (Carl Zeiss). Quantification of nuclear signal was performed with ImageJ software and statistical analysis was performed in GraphPad Prism.

### Co-immunoprecipitation

For co-immunoprecipitation experiments, cells were harvested in ice-cold PBS with 1 mM PMSF and pelleted. Pellets were lysed for 20 minutes on ice in a PBS buffer with 100 mM NaCl, 0.5% Triton, 2.5 mM EDTA, 2 mM DTT, and protease inhibitors, and then tip sonicated. After sonication lysates were clarified and loaded onto antibody beads (anti-HA agarose (Sigma) or protein G beads with hnRNP U primary antibody (Santa Cruz)) and incubated at 4 °C for 4 hours with end-over-end turning. Samples were washed with PBS with 100 mM NaCl, 0.5% Triton, 1 mM EDTA, 2 mM DTT, and protease inhibitors and then analyzed by SDS-PAGE and western blot.

### Gene expression analysis

RNA was harvested by Trizol extraction per manufacturer protocol. RT-qPCR was performed per standard methods using the following primer sequences:

KPNA1 (5′-TAGCAACATTTCTCCGCTTG-3′ and 5′TCTCTGAAT-CCCGATGAGATG-3′),

KPNA2 (5′-TGATTTTCCACATTGCTGCT-3′ and 5′-GATGATGCTACTTCTCCGCTG-3′),

KPNA3 (5′-TTTTGTTCTTCCGCAGTTCC-3′ and 5′-CGCATCAAGAGCTTCAAGAAC-3′),

KPNA4 (5′-CAACTTCATTTCGTTGTCTTCTC-3′ and 5′-CGGACAACGAGAAACTGGAC-3′),

KPNA5 (5′-CGGCATTTCTTGTTGTTGTG-3′ and 5′-TGCTGGTGACAATGCAGAAT-3′),

KPNA6 (5′-AATTGTCTTTCCCTGGGCTC-3′ and 5′-ATTGTCTACTGAAAGCTGCCG-3′), and

KPNA7 (5′-CATCGAGAAGCACTTTGGTG-3′ and 5′-GGAGGTAGGGAGCTTGGCTA-3′).

### Fluorescence polarization anisotropy

Single strand forward and reverse DNA oligonucleotides of the KPNA7 exon 7 CTCF binding motif (c.1021-1036) with and without the c.1030G > C transversion were purchased with HPLC purification (IDT DNA Technologies). Fluorescein (6-FAM) was conjugated to the 5′ end of the reverse oligo for each pair. The sequences of the oligos are as follows: WT F: 5′-ATCCAGAAGGAGGCAG-3′; WT R: 5′-/56-FAM/CTGCCTCCTTCTGGAT-3′; Mutant F: 5′-ATCCAGAAGCAGGCAG-3′; Mutant R; 5′-/56-FAM/CTGCCTGCTTCTGGAT-3′. Forward and reverse primers were annealed, and dsDNA was purified by ion exchange chromatography (QSepharose; GE Life Sciences). dsDNA was made up to10 nM in FP buffer (20 mM Tris pH 8, 200 mM KCl, 100 *μ*M ZnCl_2_, and 1 mM DTT) for fluorescence polarization (FP) experiments.

MBP-CTCF(F4-F8) was made up to 34.5 *μ*M in FP buffer and titrated with two-fold serial dilutions for both the wild-type and mutant dsDNA oligos. Oligo concentration was kept constant at 10 nM. Anisotropy measurements were made in triplicate in a black COSTAR 96-well plate (Corning Life Sciences) on a PHERAstar microplate reader (BMG Labtech) with excitation at 494 nm and emission at 525 nm at 25 °C. A binding curve was generated by fitting data to a sigmoidal modal using OriginPro Software. For the mutant oligo, which bound at a lower affinity, an apparent K_*D*_ was generated by setting the upper and lower bounds to the same as the wild-type oligo. An unpaired T-test was used to determine the significance of the difference in K_*D*_.

### Data sources

Single-cell RNA-seq of human implantation embryos data from Yan and colleagues^73^ was used to query the expression of the human *KPNA* genes. The Washington University Epigenome Browser (http://epigenomegateway.wustl.edu/browser/) was used to visualize CTCF Chip-Seq datasets from the ENCODE project^74^ with the following identifiers: ENCFF619IWL, ENCFF940XMP, ENCFF259PXQ.

### Antibodies

#### Primary

Anti-GST (B-14) – mouse monoclonal – Santa Cruz Biotechnology, sc-138; Anti-HA (16B12) – mouse monoclonal – Covance, MMS-101P; Anti-MBP – mouse monoclonal – NEB, E8032S; Anti-Imp-*β* (3E9) – mouse monoclonal – abcam, ab2811; Anti-KPNA7 – rabbit polyclonal – Millipore Sigma, HPA031395; Anti-hnRNP R – rabbit polyclonal – Millipore Sigma, SAB2700924; Anti-hnRNP U (3G6) – mouse monoclonal – Santa Cruz Biotechnology, sc-32315; Anti-Flag M2 – mouse monoclonal – Millipore Sigma, F1804; Anti-CTCF – rabbit monoclonal – Cell Signaling, D31H2.

#### Secondary

Goat anti-mouse-IRDye-800 - Rockland, 610–132- 121 (IB); Donkey anti-rabbit-Alexa Fluor-680 – Invitrogen, A10043 (IB); Goat Anti-Rabbit-Cy3 – Jackson ImmunoResearch, 115-165-144 (IF); Donkey anti-mouse-FITC – Jackson ImmunoResearch, 715-225-151 (IF); Goat anti-Mouse-Alexa Fluor-680 – Invitrogen, A21058 (IF).

## Supplementary information

Supplementary Information.

Supplementary Information.
