# Supplementary material for: An epilepsy-associated mutation in the nuclear import receptor KPNA7 reduces nuclear localization signal binding
Source: Sci Rep. 2020 Mar 16;10:4844. doi: 10.1038/s41598-020-61369-5 (PMC7076015; doi:10.1038/s41598-020-61369-5)
Supplement: Supplementary file 1 — Supplementary Information. [file 41598_2020_61369_MOESM1_ESM.pdf]

Supplementary Information:

An epilepsy-associated mutation in the nuclear import receptor KPNA7 reduces nuclear localization signal binding

Luke T. Oostdyk[1,2], Zhenjia Wang[3], Chongzhi Zang[3], Hui Li[1,4], Michael J. McConnell[1,5,6], and Bryce M. Paschal[1,2*]

[1]Department of Biochemistry & Molecular Genetics, University of Virginia School of Medicine, Charlottesville, VA, 22908; [2]Center for Cell Signaling, University of Virginia School of Medicine, Charlottesville, VA, 22908; [3]Center for Public Health Genomics and Department of Public Health Sciences, University of Virginia School of Medicine, Charlottesville, VA, 22908; [4]Department of Pathology, University of Virginia School of Medicine, Charlottesville, VA 22908; [5]Center for Brain Immunology and Glia, University of Virginia School of Medicine, Charlottesville, VA 22908, USA; [6]Department of Neuroscience, University of Virginia School of Medicine, Charlottesville, VA, 22908

* To whom correspondence should be addressed: Bryce M. Paschal: Department of Biochemistry & Molecular Genetics, University of Virginia, Charlottesville, VA, 22908; paschal@virginia.edu

a

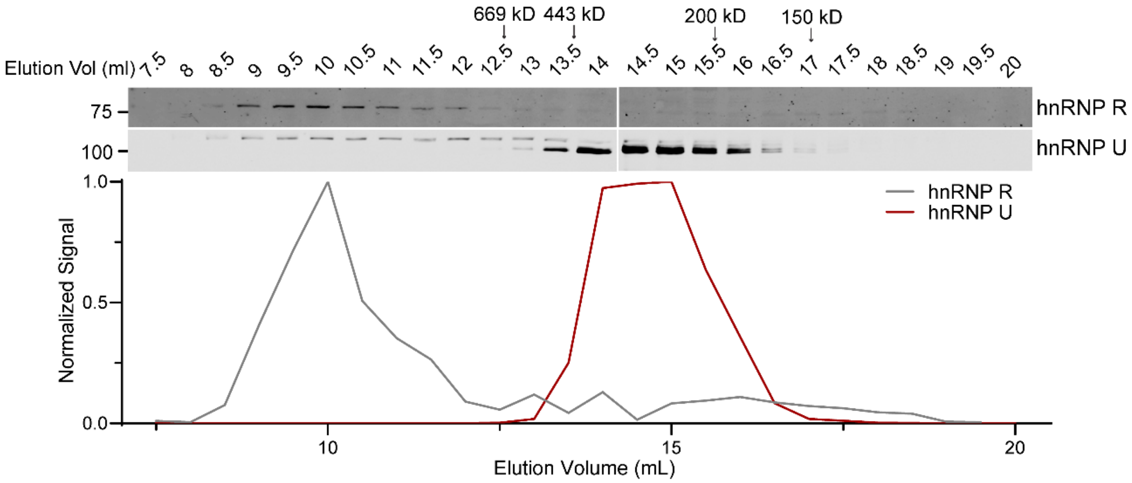

Figure S1: Size exclusion chromatography analysis of hnRNP proteins. **(a)** HEK293T cell lysate was analyzed by size exclusion chromatography with a Superose6 column. Fractions were analyzed by SDS-PAGE and western blot analysis. Bands corresponding to hnRNP R and hnRNP U were quantified and plotted. Elution volume of standards of the indicated size are indicated above the blots.

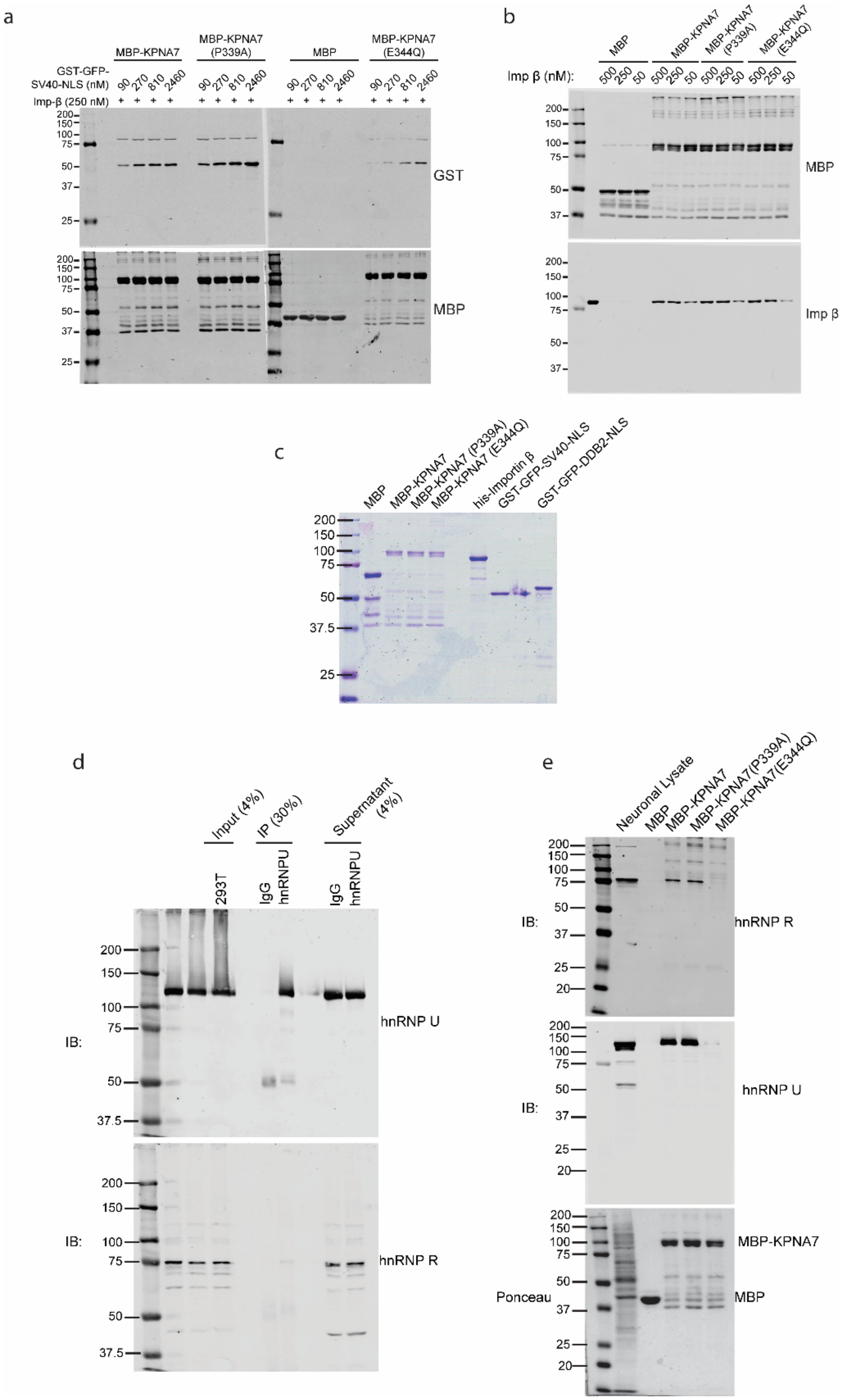

Figure S2: Full-size gels and blots from indicated panels: **(a)** Figure 1b, **(b)** Figure 1c, **(c)** Figure 2b, **(d)** Figure 3d and **(e)** Figure 3e.

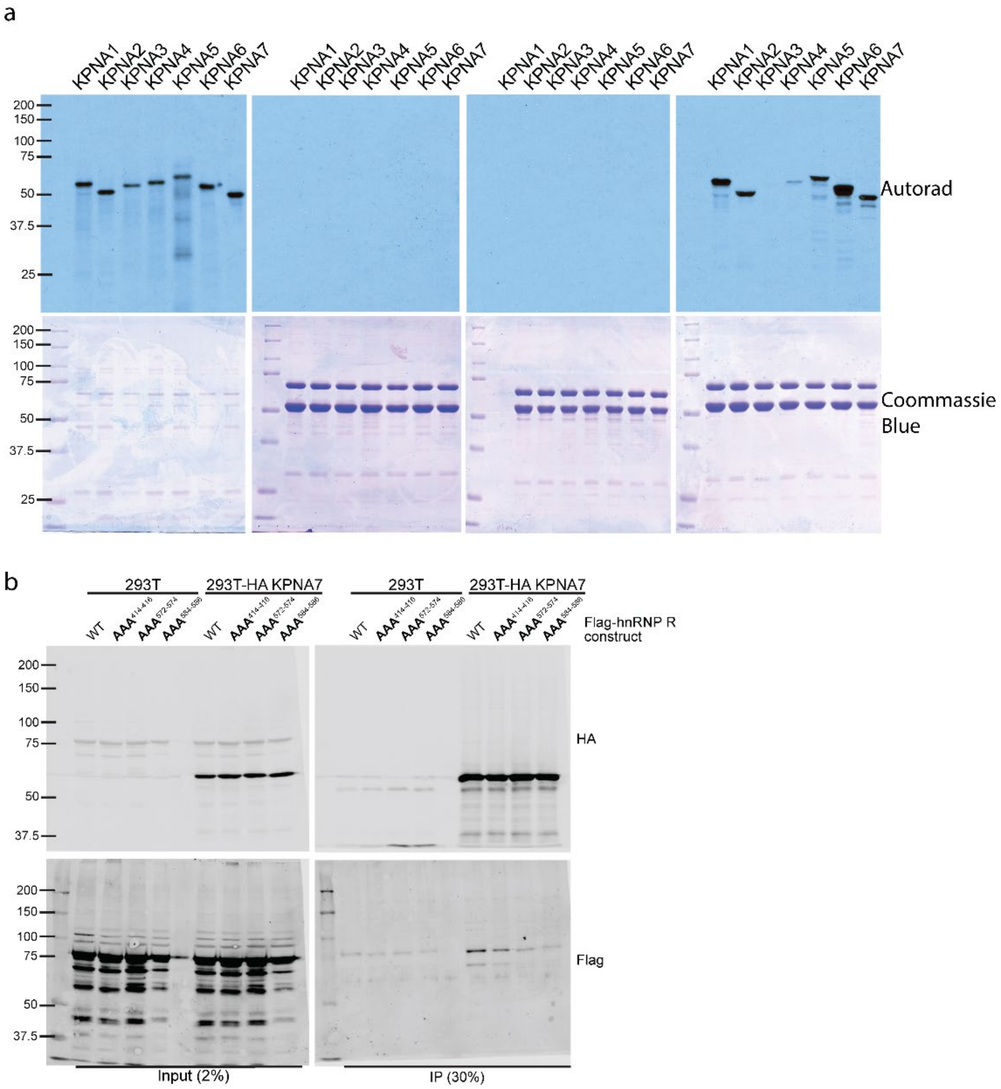

Figure S3: Full-size gels and blots from indicated panels: **(a)** Figure 4e and **(b)** Figure 4f.

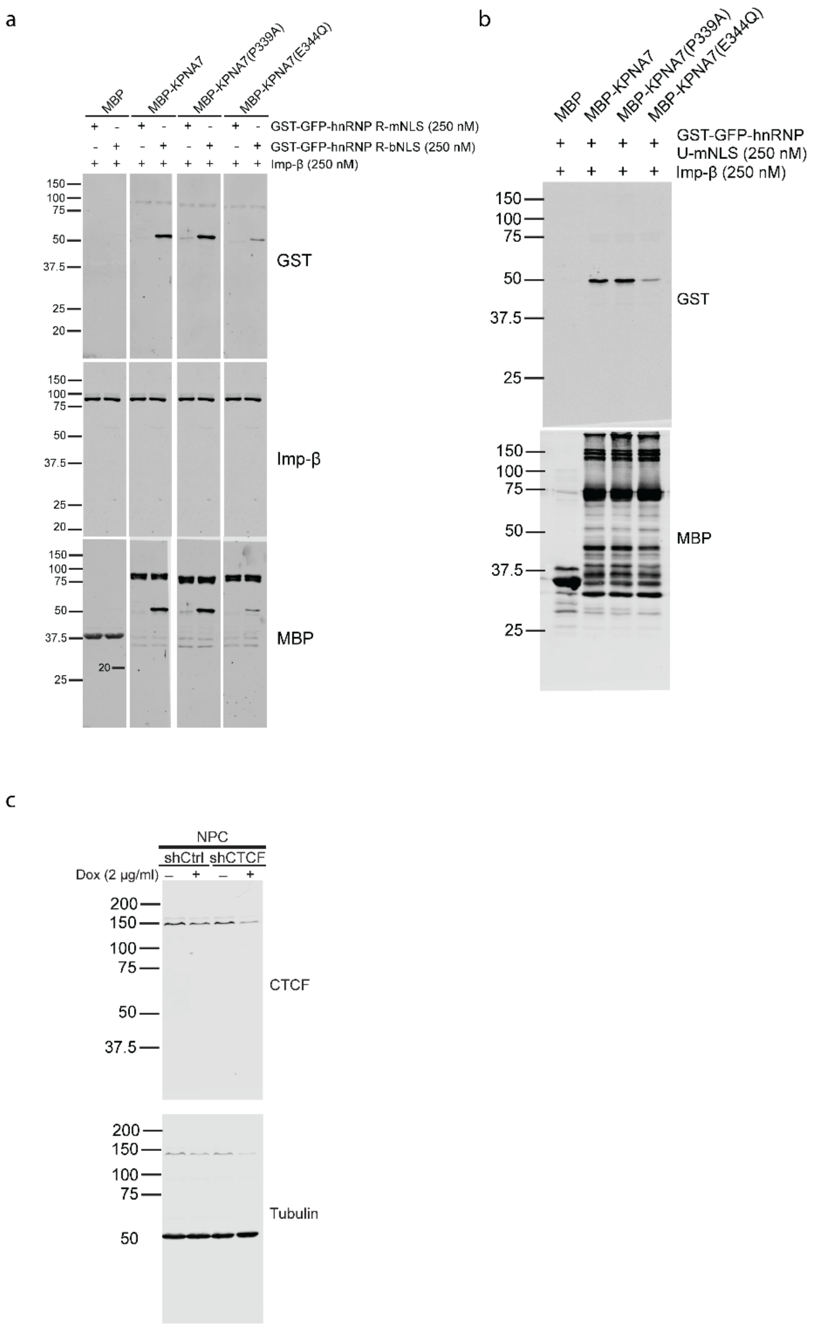

Figure S4: Full-size gels and blots from indicated panels: **(a)** Figure 5a, **(b)** Figure 6b and **(c)** Figure 10a.

Supplementary Table 2: KPNA7 interacting proteins identified by SILAC-MS with over 10 identified peptides and abundance ratios greater than 2.75

Description	Gene Symbol	# PSMs	Abundance Ratio: (F1,Heavy) /(F1,Light)
Isoform 2 of Heterogeneous nuclear ribonucleoprotein R OS=Homo sapiens OX=9606 GN=HNRNPR	HNRNPR	92	100
Isoform 2 of Serine/threonine-protein phosphatase PP1-alpha catalytic subunit OS=Homo sapiens OX=9606 GN=PPP1CA	PPP1CA	11	23.567
Pleiotrophin OS=Homo sapiens OX=9606 GN=PTN PE=1 SV=1	PTN	12	4.607
Histone deacetylase complex subunit SAP18 OS=Homo sapiens OX=9606 GN=SAP18 PE=1 SV=1	SAP18	36	4.017
RNA-binding protein with serine-rich domain 1 OS=Homo sapiens OX=9606 GN=RNPS1 PE=1 SV=1	RNPS1	38	3.99
Protein polybromo-1 OS=Homo sapiens OX=9606 GN=PBRM1 PE=1 SV=1	PBRM1	57	3.79
Cyclin-dependent kinase 11B OS=Homo sapiens OX=9606 GN=CDK11B PE=1 SV=4	CDK11B	24	3.531
Isoform SM-B1 of Small nuclear ribonucleoprotein-associated proteins B and B' OS=Homo sapiens OX=9606 GN=SNRPB	SNRPB	21	3.53
A-kinase anchor protein 17A OS=Homo sapiens OX=9606 GN=AKAP17A PE=1 SV=2	AKAP17A	23	3.384
Bromodomain-containing protein 9 OS=Homo sapiens OX=9606 GN=BRD9 PE=1 SV=2	BRD9	13	3.358
Splicing factor, arginine/serine-rich 19 OS=Homo sapiens OX=9606 GN=SCAF1 PE=1 SV=3	SCAF1	15	3.338
RNA-binding motif protein, X chromosome OS=Homo sapiens OX=9606 GN=RBMX PE=1 SV=3	RBMX	87	3.189
Bromodomain-containing protein 7 OS=Homo sapiens OX=9606 GN=BRD7 PE=1 SV=1	BRD7	12	3.183
Heterogeneous nuclear ribonucleoprotein U OS=Homo sapiens OX=9606 GN=HNRNPU PE=1 SV=6	HNRNPU	442	3.159
Pre-mRNA-splicing factor 38B OS=Homo sapiens OX=9606 GN=PRPF38B PE=1 SV=1	PRPF38B	15	3.126
U2 snRNP-associated SURP motif-containing protein OS=Homo sapiens OX=9606 GN=U2SURP PE=1 SV=2	U2SURP	67	3.124
Isoform 2 of Integrator complex subunit 3 OS=Homo sapiens OX=9606 GN=INTS3	INTS3	11	3.068
Zinc finger protein 768 OS=Homo sapiens OX=9606 GN=ZNF768 PE=1 SV=2	ZNF768	13	3.062
Peptidyl-prolyl cis-trans isomerase G OS=Homo sapiens OX=9606 GN=PPIG PE=1 SV=2	PPIG	24	3.061
40S ribosomal protein S11 OS=Homo sapiens OX=9606 GN=RPS11 PE=1 SV=3	RPS11	11	3.035
FACT complex subunit SSRP1 OS=Homo sapiens OX=9606 GN=SSRP1 PE=1 SV=1	SSRP1	10	3.014
Pinin OS=Homo sapiens OX=9606 GN=PNN PE=1 SV=5	PNN	57	2.934
E3 ubiquitin-protein ligase Hakai OS=Homo sapiens OX=9606 GN=CBLL1 PE=1 SV=1	CBLL1	10	2.916
Pre-mRNA-processing factor 40 homolog A OS=Homo sapiens OX=9606 GN=PRPF40A PE=1 SV=2	PRPF40A	86	2.912
Serine/arginine-rich splicing factor 9 OS=Homo sapiens OX=9606 GN=SRSF9 PE=1 SV=1	SRSF9	31	2.902
Cleavage and polyadenylation specificity factor subunit 2 OS=Homo sapiens OX=9606 GN=CPSF2 PE=1 SV=2	CPSF2	26	2.9
Protein virilizer homolog OS=Homo sapiens OX=9606 GN=VIRMA PE=1 SV=2	KIAA1429	32	2.887
Splicing factor U2AF 35 kDa subunit OS=Homo sapiens OX=9606 GN=U2AF1 PE=1 SV=3	U2AF1	95	2.883
Isoform 4 of Apoptotic chromatin condensation inducer in the nucleus OS=Homo sapiens OX=9606 GN=ACIN1	ACIN1	126	2.881
Nucleolar protein 4 OS=Homo sapiens OX=9606 GN=NOL4 PE=1 SV=2	NOL4	12	2.859
General transcription factor IIF subunit 2 OS=Homo sapiens OX=9606 GN=GTF2F2 PE=1 SV=2	GTF2F2	27	2.849

U4/U6.U5 tri-snRNP-associated protein 2 OS=Homo sapiens OX=9606 GN=USP39 PE=1 SV=2	USP39	20	2.816
Transcription elongation factor SPT5 OS=Homo sapiens OX=9606 GN=SUPT5H PE=1 SV=1	SUPT5H	36	2.797
Pre-mRNA-splicing factor 38A OS=Homo sapiens OX=9606 GN=PRPF38A PE=1 SV=1	PRPF38A	11	2.795
RNA-binding protein 39 OS=Homo sapiens OX=9606 GN=RBM39 PE=1 SV=2	RBM39	59	2.779
Isoform 2 of Heterogeneous nuclear ribonucleoprotein U OS=Homo sapiens OX=9606 GN=HNRNPU	HNRNPU	393	2.774
Protein DEK OS=Homo sapiens OX=9606 GN=DEK PE=1 SV=1	DEK	60	2.774
Zinc finger RNA-binding protein OS=Homo sapiens OX=9606 GN=ZFR PE=1 SV=2	ZFR	10	2.76
60S ribosomal protein L24 OS=Homo sapiens OX=9606 GN=RPL24 PE=1 SV=1	RPL24	10	2.754
SAP30-binding protein (Fragment) OS=Homo sapiens OX=9606 GN=SAP30BP PE=1 SV=1	SAP30BP	14	2.75
